# Folic acid supplementation regulates key immunity-associated genes and pathways during the periparturient period in dairy cows

**DOI:** 10.5713/ajas.18.0852

**Published:** 2019-04-15

**Authors:** Muhammad Zahoor Khan, Zhichao Zhang, Lei Liu, Di Wang, Siyuan Mi, Xueqin Liu, Gang Liu, Gang Guo, Xizhi Li, Yachun Wang, Ying Yu

**Affiliations:** 1 Key Laboratory of Animal Genetics, Breeding, and Reproduction, Ministry of Agriculture & National Engineering Laboratory for Animal Breeding, College of Animal Science and Technology, China Agricultural University, Beijing 100193, China; 2 Hebei Shoulon Modern Agricultural Science and Technology Co. Ltd., Dingzhou 073000, China; 3 Beijing Sanyuan Breeding Technology Co. Ltd., Capital Agribusiness Group, Beijing 100193, China

**Keywords:** Folic Acid, Periparturient Dairy Cow, Transcriptome, Anti-inflammation, Immunity

## Abstract

**Objective:**

The current research was aimed to profile the transcriptomic picture of the peripheral blood lymphocytes (PBLs) associated with immunity in Chinese Holsteins supplemented orally with coated folic acid during the periparturient period.

**Methods:**

The total of 123 perinatal cows were selected for this study and divided into three groups; group A (n = 41, 240 mg/500 kg cow/d), group B (n = 40, 120 mg/500 kg cow/d) and group C (n = 42, 0 mg/cow/d) based on the quantity of folic acid fed. Three samples of PBLs were selected from each folic acid treated group (high, low, and control) and RNA sequencing method was carried out for transcriptomic analysis.

**Results:**

The analysis revealed that a higher number of genes and pathways were regulated in response to high and low folic acid supplementation compared to the controls. We reported the novel pathways tumor necrosis factor (TNF) signaling, antigen processing and presentation, *Staphylococcus aureus* infection and nuclear factor (NF)-kappa B signaling pathways) and the key genes (e.g. C-X-C motif chemokine ligand 10, TNF receptor superfamily member 1A, cluster difference 4, major histocompatibility complex, class II, DQ beta, NF-kappa-B inhibitor alpha, and TNF superfamily 13) having great importance in immunity and anti-inflammation in the periparturient cows in response to coated folic acid treatment.

**Conclusion:**

Collectively, our study profiled first-time transcriptomic analysis of bovine lymphocytes and compared the involved cytokines, genes, and pathways between high vs control and low vs control. Our data suggest that the low folic acid supplementation (120 mg/500 kg) could be a good choice to boost appropriate immunity and anti-inflammation as well as might being applied to the health improvement of perinatal dairy cows.

## INTRODUCTION

Folate plays vital roles in cell growth and proliferation through regulating the synthesis and stabilization of deoxyribonucleic acid (DNA) and ribonucleic acid (RNA), and its deficiency may lead to many serious diseases [1]. Moreover, the adequate levels of folate are crucial for the proper immune function, particularly during pregnancy. Ruminants synthesize folic acid due to bacteria in their rumen [2]. In some conditions like the periparturient period, the dairy cows experience severe immune and metabolic depression due to pregnancy stress, which increases their susceptibility to mastitis and other infectious diseases [3]. Interestingly, the secretion of folates is also increased six times greater in colostrum than in milk 39 days post-calving [4]. Whether the demand for folic acid may increase or not for maintenance of immune system capacity during this period is still undefined.

The physiological responses of an individual to the environment and stress are driven by the modulation of its genes through the production of mRNAs or transcriptome [5]. A combined supplement of vitamins B_9_ and B_12_ by intramuscular route significantly regulates the hepatic and mammary gland gene expression in lactating dairy cows [6] and also facilitates the lactational performance and energy status in multiparous cows [7]. Moreover, Graulet et al [8], also reported that the intramuscular injection of folic acid facilitates metabolic efficiency and increases the milk production ability of dairy cows [8]. However, the oral supplementation of coated folic acid is widely ignored particularly during the perinatal period in dairy cows. It is essential that the ruminant’s folate requirements must be adjusted particularly during the periparturient period to avoid any loss in production ability and to prevent the metabolic or infectious diseases.

To address whether the supplementation of folic acid is needed for health maintenance and animal production activities especially during the perinatal time in a dairy cow, we conducted first a molecular-based study, in which the influence of coated folic acid supplementation on the immune status of Holstein cows was tested. By taking the advantage of RNA-seq assay, we evaluated the transcriptomic profiles of bovine peripheral blood lymphocytes (PBLs) for immunity-associated genes and pathways in response to oral supplementation of coated folic acid.

## MATERIALS AND METHODS

### Sample population and folic acid treatment

**Figure 1:**
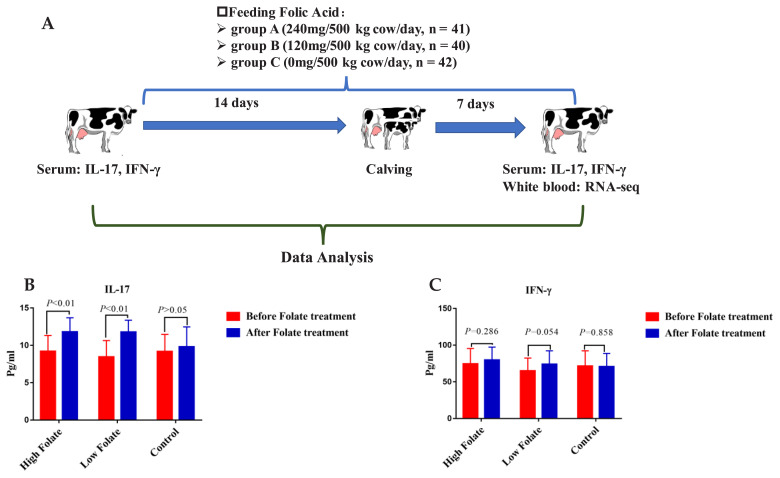
The effects of folic acid supplementation on serum cytokines of transition dairy cows. (A) Experimental procedures of the entire study (B) The level of interleukin 17 (IL-17) (pg/mL) and (C) interferon-gamma (IFN-γ) (pg/mL) in Chinese Holstein cow’s pre and post-supplementation of folic acid.

A total of 123 perinatal cows having similar parity, weight and prenatal period were selected and divided into three groups; group A (n = 41, 240 mg/500 kg cow/d), group B (n = 40, 120 mg/500 kg cow/d) and group C (n = 42, 0 mg/cow/d) based on the quantity of folic acid fed during the perinatal stage. The folic acid treatment lasted 21 days, including 14 days before calving till seven days after the calving (Figure 1A). As the amount of folic acid supplementation was small (mg level), it was necessary to mix it with a certain amount of palletized concentrate to ensure enough folic acid was supplied to each cow.

### Sample collection

Blood samples were collected from each group of Chinese Holsteins treated with folic acid before the treatment and seven days post-calving. The blood samples were taken in coagulant tubes, kept for 10 minutes at room temperature and then centrifuged at 12,000 rpm/15 m. Furthermore, serum was collected and stored at −20°C and sent to Beijing Huaying Biological Technology Research Institute for the detection of serum cytokines interleukin 17 (IL-17) and interferon-gamma (IFN-γ) by performing radioimmunoassay (Sino-UK, Beijing, China). In brief, each serum sample was initially centrifuged at 3,000 rpm/min for 5 min at 4°C. Next, 100 μL “cold” antigen (supernatant), 100 μL antibody and 100 μL “hot” antigen (radiolabeled antigen, 125-I) were mixed thoroughly and kept at 4°C for 24 h. Then, 500 μL separating buffer was added and mixed well. The mixture stayed at room temperature for 20 min. Later, the mixture was centrifuged at 3,500 rpm/min for 25 min at 4°C. Finally, the supernatant was discarded, and the precipitate bound with antigen was used to measure the radioactivity by a gamma counter.

In addition, the white blood cells at seven days post-calving were collected into a tube containing one mL Trizol for RNA extraction.

### Extraction of RNA and sample preparation

Total RNA was isolated from white blood cells using the TRIzol reagent (Invitrogen, Carlsbad, CA, USA). Isolated RNA was purified by removing DNA through DNase I digestion (Qiagen, Heldon, Germany). RNA degradation and contamination were detected by 1% agarose gels. The RNA concentration was assessed using NanoPhotometer spectrophotometer (Implen, Westlake, CA, USA). The extracted RNA was stored at −80°C and a whole nine samples (three from each group) were sent to the company (Annoroad Gene Technology Co., Ltd, Beijing, China) for RNA sequencing.

### Library preparation for RNA-Seq

Three samples for each group were selected for library preparation. A total amount of 2 μg RNA per sample was used as input material for the RNA sample preparations. Sequencing libraries were generated using NEBNext Ultra RNA Library Prep Kit for Illumina (#E7530L, NEB, Ipswich, MA, USA) following the manufacturer’s recommendations and index codes were added to attribute sequences to each sample. Briefly, mRNA was purified from total RNA using poly-T oligo-attached magnetic beads. Fragmentation was carried out using divalent cations under elevated temperature in NEBNext first-strand synthesis reaction buffer (5×). First strand cDNA was synthesized using random hexamer primer and RNase H. Second strand cDNA synthesis was subsequently performed using a buffer, dNTPs, DNA polymerase I and RNase H. The library fragments were purified with QiaQuick PCR kits and elution with elution buffer, then terminal repair A-tailing and adapter added were implemented. The aimed products were retrieved by agarose gel electrophoresis, and polymerase chain reaction (PCR) was performed, then the library was completed.

### Library examination

RNA concentration of library was measured using Qubit RNA Assay Kit in Qubit 3.0 to preliminary quantify and then dilute to 1 ng/μL. Insert size was assessed using the Agilent Bioanalyzer 2100 system (Agilent Technologies, Santa Clara, CA, USA), and qualified insert size was accurately quantified using Step One Plus Real-Time PCR System (Library valid concentration >10 nM). The clustering of the index-coded samples was performed on a cBot cluster generation system using HiSeq PE Cluster Kit v4-cBot-HS (Illumina, USA) according to the manufacturer’s instructions. After cluster generation, the libraries were sequenced on an Illumina Hiseq 4000 platform, and 150 bp paired-end reads were generated.

### Bioinformatics analysis for RNA-Seq

Reads obtained from the sequencing machine include raw reads containing adapters or low quality bases, which affect the following assembly and analysis. To get high quality reads, the data was filtered to remove adaptor sequences, unknown nucleotides (N), low-quality reads having more than 50% of low quality (Q<20) bases, using next generation sequencing quality control toolkit version 2.3.3. A set of genomic index files of reference genome was built using Bowtie version 2.2.5, and clean reads were mapped to the Bos taurus reference genome (UMD3.1) obtained from Ensembl (ftp://ftp.ensembl.org/pub/release-73/fasta/bos_taurus/dna/) using Tophat version 2.1.0 [9]. Then, the transcripts were assembled using Cufflinks version 2.2.1. Transcript files generated by Cufflinks were added to a single-merged transcriptome annotation using Cuffmerge version 2.2.1. differentially expressed genes (DEGs) and transcripts were identified between different sample groups using Cuffdiff version 2.2.1 [10]. Gene expression values were calculated by counting the number of fragments per kilobase of transcript per million mapped fragments, and Cuffdiff was applied to measure significant differences among the three groups.

Biological process of gene ontology (GO) enrichment and Kyoto encyclopedia of genes and genomes (KEGG) pathway analyses were conducted with DEGs using the database for annotation, visualization and integrated discovery (DAVID) pathways analysis tool (http://david.abcc.ncifcrf.gov/). The DEGs were administered into STRING v10 to generate a protein-protein interaction (PPI) network and to predict physical/functional PPIs.

### Quantitative reverse transcription polymerase chain reaction validation for RNA-seq analysis

To verify the gene expression data gained by RNA sequencing analysis, quantitative reverse transcription PCR (RT-qPCR) was carried out on eight randomly selected DEGs namely mitogen-activated protein kinase 13 (*MAPK13*), cluster difference 4 (*CD4*), selectin P ligand (*SELPLG*), C-X-C motif chemokine receptor 2 (*CXCR2*), major histocompatibility complex antigen class II (*BLA-DQB*), C-C motif chemokine ligand 5 (*CCL5*), NF-kappa-B inhibitor alpha (*NFKBIA*), and TNF superfamily 13 (*TNFSF13*) using the total RNA used for RNA-seq. The primers were designed for all the targeted genes by using Primer5 software and were further validated through Oligo 6.0. The detail of the primers has been given in the supplementary material (Supplementary file 1). The cDNA was prepared using PrimeScriptTM RT reagent kit according to the manufacturer’s instructions (Takara, Dalian, China). The RT-qPCR reactions were carried out in a final volume of 20 μL with the Roche SYBR Green PCR Kit (Roche, Hercules, CA, USA) according to the manufacturer’s protocol. The bovine housekeeping glyceraldehyde 3-phosphate dehydrogenase was used as the internal standard to adjust the input of cDNA and to normalize the expression of target genes. Duplicate RT-qPCRs were performed on each cDNA, and the average Ct value was used for further analyses.

### Statistical analysis

SAS9.2 general linear model program was used for statistical analysis, the folic acid effect model was analyzed as follows;

Model: y=μ+α+β+γ+e

Among them: *y*, cytokines; *μ*, population mean; *α*, parity effect; *β*, weight effect; *γ*, folic acid effect; *e*, random residuals.

Furthermore, Student’s *t*-test was used to assess the difference between pre and post-folic acid treated groups for cytokines IL-17 and IFN-γ as well as to analyze the RT-qPCR results.

## RESULTS

### Effects of folic acid supplementation on serum cytokines during the periparturient period in Chinese Holstein

In this study, the levels of serum cytokines IL-17 and IFN-γ were measured for each sample before and after folic acid supplementation. By analyzing with the statistical model, we found that before folic acid addition there were no significant differences for the two cytokines among high (240 mg/500 kg body weight), low (120 mg/500 kg body weight) folate supplemented groups and control group (0 mg/500 kg body weight) (p<0.05, Supplementary file 1; Figure 1). It indicates that the selection and grouping of the samples in this study was representative.

Furthermore, *t*-test analyses showed that folic acid supplementation for 21 days considerably increased the level of IL-17 in the serum of high and low folate groups, which is significantly higher than the pre-folic acid treated group (p<0.05, Figure 1B). Similarly, the IFN-γ was moderately up-regulated by low folic acid treatment (p = 0.054, Figure 1C). There was no significant difference in the control cows post-folic acid supplementation for the two cytokines. These findings imply that the addition of folic acid as a nutrient during the periparturient period has an apparent impact on the regulation of the IL-17 and IFN-γ, which is necessary for the maintenance of proper immune function in periparturient dairy cows. To get more clear proof of folate influence during the transition period in dairy cattle, we extended our study by conducting a transcriptomic analysis for PBLs.

### Comparison of differentially expressed genes among the three groups (high vs control, low vs control, and high vs low)

**Figure 2:**
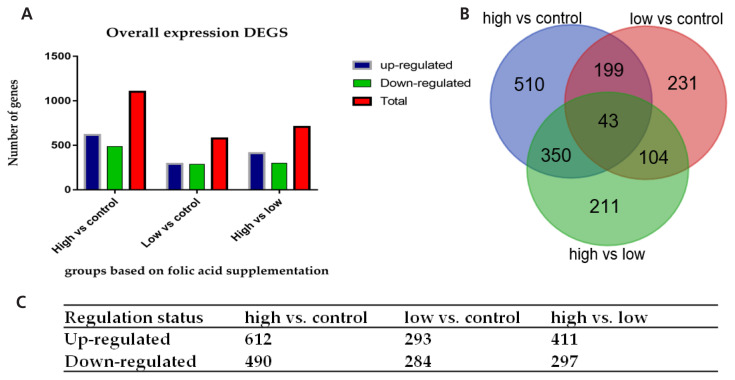
Overall DEGs in the three comparisons (high vs control, low vs control, and high vs low). (A) The graphical presentation of a total number of DEGs in the three comparisons. (B) Genes commonly shared among the three comparisons. (C) The number of up-regulated and down-regulated DEGs in each comparison. DEGs, differentially expressed genes.

Comparing genome-wide transcripts levels in the Chinese Holstein cows supplemented with High folate and control cows, we identified 1,102 DEGs, meeting the criteria of p<0.05. Among these genes, 612 (55.5%) were up-regulated, and 490 (44.5%) were down-regulated in the high folic acid supplemented group. In addition, 577 DEGs were detected in the cows supplemented with a low level of folic acid (group low, 120 mg/500 kg) compared with the control using the criteria of p<0.05. Among these genes, 293 (50.8%) were up-regulated, and 284 (49.2%) were down-regulated in group Low folic acid. The total numbers of DEGs detected in high vs low was 708, out of which 297 (41.9%) were down-regulated, and 411 (58.1%) were detected to be up-regulated (Figure 2A, 2C). Furthermore, the Venn diagram reveals that 43 genes were found to be commonly distributed in the three comparisons of high vs control, low vs control, and high vs low folic acid supplemented groups (Figure 2B).

### Significant pathways in response to high and low folic acid supplementation

For an increased understanding of signaling pathways mediated by the folate treatment, the identified DEGs in the three comparisons were subjected to pathway analysis with Kyoto encyclopedia of genes and genomes (KEGG).

### High vs control comparison

**Figure 3:**
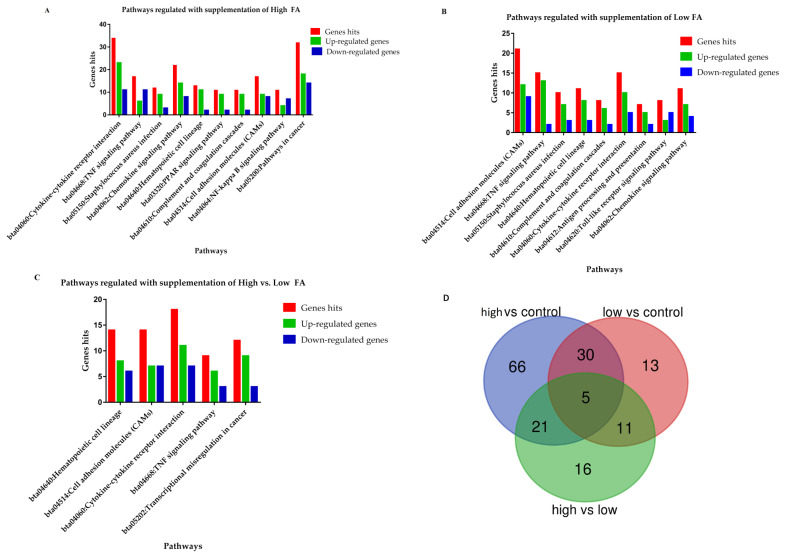
The graphical presentations of biological pathways involved in response to folic acid treatments. (A and B) The immunity associated pathways mediated in response to high and low folic acid treatments separately. (C) The immunity associated pathways in the comparison of high vs low. (D) The Venn diagram showed the distributions and shared genes among the three comparisons.

Combined with enrichment analysis, 40 KEGG pathways were reported in this comparison (Supplementary file 2), of which 32 were significantly (p<0.05) regulated while the rest of the eight did not reach to the significant level (p>0.05). Furthermore, ten significantly mediated immunity-linked pathways (Table 1) and the genes distributed in each pathway are enlisted in Supplementary file 3. The number of up and down-regulated DEGs involved in the ten immunity-linked KEGG pathways are presented in Figure 3A.

### Low vs control comparison

Total of 23 pathways were enriched in response to low folic acid supplementation, out of them, 19 were significantly mediated (Supplementary file 2). The nine pathways which have key roles in the immunity regulation of the body were selected from the 19 significantly associated pathways and listed in Table 1. Between the comparisons of high vs control and low vs control, seven commonly shared pathways were reported, furthermore our findings revealed that most of the DEGs among these pathways were up-regulated (Figure 3B; Supplementary file 3).

### High vs low comparison

Thirteen KEGG pathways were enriched in this pathway, of which 11 reach a significant level (p<0.05) (Supplementary file 2). Additionally, five having a key role in immunity and anti-inflammation were documented (Table 1; Figure 3C). Additionally, it was found that the reported DEGs were almost equally distributed in all the five’s biological function pathways (Supplementary file 3).

Taking together, four out of 12 (33%) pathways were shared in all the three comparisons, including cytokine-cytokine receptor interaction, tumor necrosis factor (TNF) signaling pathway, hematopoietic cell lineage, and cell adhesion molecules pathways.

### Commonly shared genes among all the pathways of the three comparisons

The total of 125, 59, and 53 DEGs were documented in the three comparisons of high vs control, low vs control, and high vs low, respectively. Out of these DEGs, five genes namely matrix metallopeptidase 9 (*MMP9*), *CCL5*, *CD24*, C-X3-C motif chemokine receptor 1 (*CX3CR1*), and major histocompatibility complex, class II, DQ alpha 5 (*BoLA-DQA5*) were commonly shared among the three comparisons. Furthermore, 30, 11, and 21 DEGs were found to be shared between high vs control and low vs control, low vs control and high vs low, as well as high vs low and high vs control, respectively (Figure 3D; Supplementary file 4).

### Functional analysis of peripheral blood lymphocytes transcriptional changes in response to folic acid supplementation

To gain new insights into the underlying functions of the DEGs in the three comparisons, we used GO to analyze the identified DEGs, which is a well-documented and widely used annotation system that assigns molecular function, biological process, and cellular component information to gene products.

### High vs control comparison

**Figure 4:**
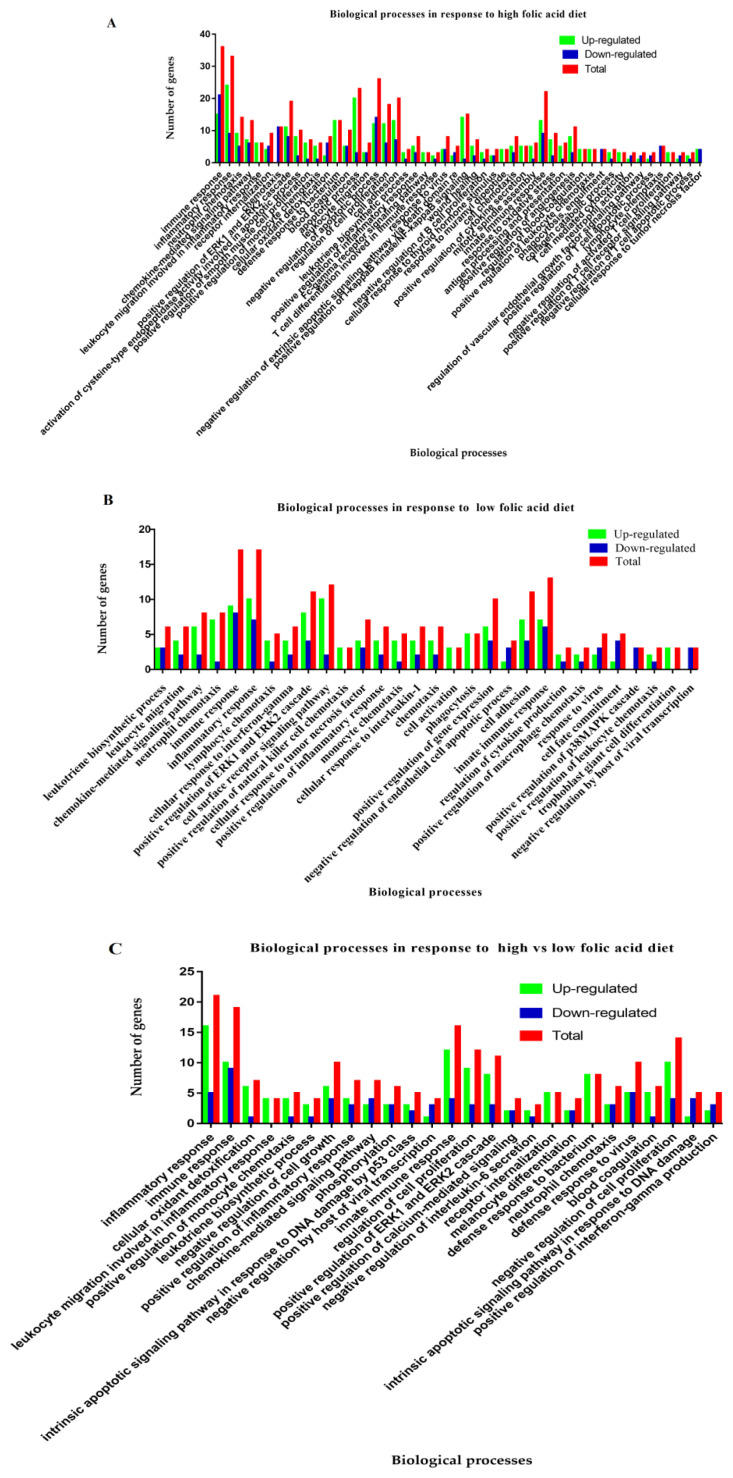
The graphical presentation of biological function processes in response to folic acid supplementation in Holsteins. (A) High vs control. (B) Low vs control. (C) High vs low.

A total of 104 different processes related to biological function were regulated in response to high folic acid diet (Supplementary file 5). Out of these, 63 biological function processes attained a significant level (p<0.05). Among these 63 significant biological processes, 50 were associated with immunity development. Furthermore, a total of 554 DEGs (204 down-regulated, i.e., mitogen-activated protein kinase kinase kinase 8 (*MAP3K8*), interleukin 2 receptor subunit beta, chemokine ligand 8 (*CCL8*) and 350 up-regulated, i.e., myeloid differentiation primary response 88 (*MYD88*), complement component 5a receptor 1 (*C5AR1*), BCL3 transcription coactivator (*BCL3*), and lipoprotein lipase (*LPL*) were involved in the biological function processes (Supplementary file 5). The graphs of all the biological processes involved are shown in Figure 4A and Supplementary file 6. Additionally, we observed that DEGs related to metabolism were successfully regulated by high folic acid diet as shown in Supplementary file 5.

### Low vs control comparison

By using GO analysis, it was shown that the DEGs regulated a total of 77 biological function processes in low vs control folic acid treated group. Out of them, 40 showed a significant difference (p<0.05) (Supplementary file 5), among them 30 biological function processes were closely associated with immune and inflammatory responses (Figure 4B). The total of 258 DEGs (90 down-regulated, i.e., *CCL5*, KIT proto-oncogene receptor tyrosine kinase (*KIT*), *NFKBIA*, Fos proto-oncogene, AP-1 transcription factor subunit (*FOS*), and 168 up-regulated, i.e., C-X-C motif chemokine ligand 10 (*CXCL10*), platelet activating factor receptor (*PTAFR*), *BLA-DQB*, and *CD4* were significantly involved in the biological function processes. The results showed that 120 mg folic acid addition per cow per day during the transition period is also beneficial for improving immunity, inflammatory and metabolic associated biological function processes (Supplementary file 6).

### High vs low comparison

Comparing transcript levels in the Chinese Holstein cows fed with a high and low intake folic acid, we identified 708 DEGs, meeting the criteria of p<0.05. Out of them, 255 DEGs were found to be in biological function categories, and the expression status showed that 163 DEGs were up-regulated i.e., TNF receptor superfamily member 1A (*TNFRSF1A*), *CD14*, colony stimulating factor 1 receptor (*CSFIR*) and 92 were detected to be down-regulated i.e., *CCL4*, arachidonate 5-lipoxygenase (*ALOX5*), and BRCA1 DNA repair associated (*BRCA1*), in the biological function categories (Figure 4C). The total detected biological processes were 61 in the comparison of high vs low, out of them, 32 were significantly regulated (Supplementary file 5). Of which 26 processes were linked to immunity and inflammatory responses (Figure 4C). It was noticed that folic acid also mediated metabolic-related biological function processes which reveal their multiple roles during the periparturient period (Supplementary file 6).

### Protein-protein interaction networks of differentially expressed genes significantly enriched in the immunity associated pathways

**Figure 5:**
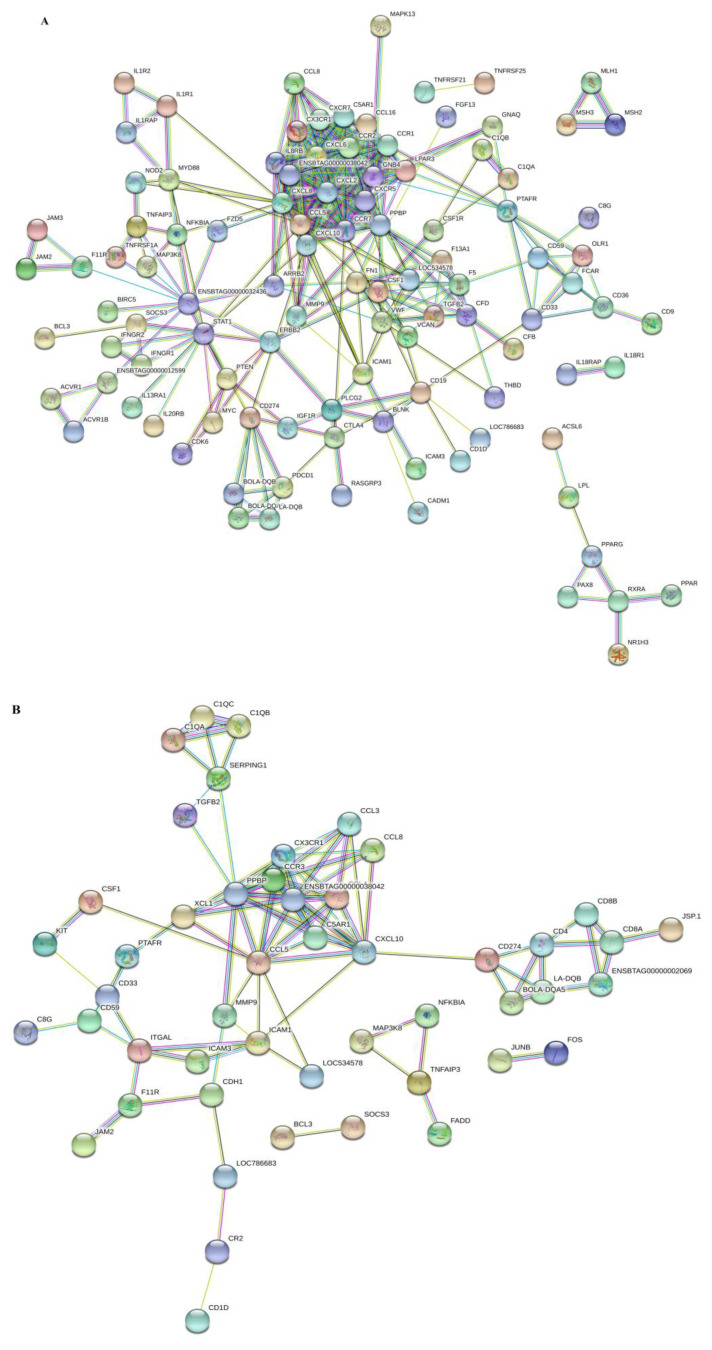
Protein-protein interaction (PPI) networks in the comparison of high vs control (A) and low vs control (B), with the confidence level of 0.7. Various colour lines represent seven types of evidence used in predicting associations. Red line, fusion evidence; blue line, co-occurrence evidence; yellow line, text mining evidence; green line: neighbourhood evidence; purple line, experimental evidence; light blue line, database evidence; and the black line, co-expression evidence.

To further evaluate the interconnection among DEGs detected in immune-related pathways, we analyzed the corresponding proteins of the DEGs through String analysis in the comparisons of high vs control and low vs control, with the confidence level of 0.7. The PPI networking analysis revealed that most of the proteins in high vs control are highly interconnected (Figure 5A). However, the PPI networks obtained from low vs control showed that the proteins ratio interconnection with each other was lower compared to high vs control. Keeping in view, the PPI in response to high folic acid treatment showed that CCL5, CXCL6, CXCR2, chemokine receptor 1 (CCRI), CCL16, retinoid X receptor alpha, SMAD family member 3, MYC proto-oncogene, bHLH transcription factor, MAP3K8, interleukin 8 receptor, beta, signal transducer and activator of transcription 1 (STAT1), CXCR5, NFKBIA, lysophosphatidic acid receptor 3 (LPAR3), MMP9, major histocompatibility complex, class II, DQ alpha 1, arrestin beta 2, and C-C motif chemokine 3 (LOC525415) distributed in the central parts. While in low folic acid supplemented cows, PPI network revealed that JunB proto-oncogene, AP-1 transcription factor subunit (JUNB), FOS, CCL5, CCL3, MHC class I JSP.1 (JSP.1), flavin adenine dinucleotide (FAD), CD4, and CXCL10 have the central positions and highly interconnected with various kinds of proteins (Figure 5B).

### Verification of RNA sequencing results by using quantitative reverse transcription polymerase chain reaction

**Figure 6:**
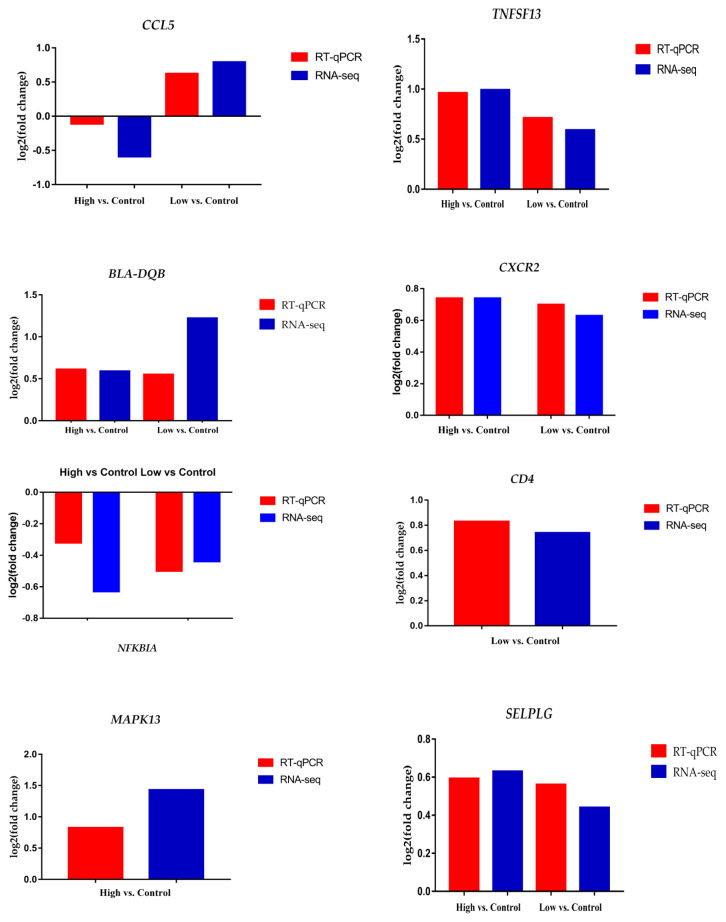
Validation of RNA-seq data analysis using RT-qPCR. The fold changes of genes *CCL5*, *TNFSF13*, *BLA-DQB*, *CXCR2*, *NFKBIA*, *CD4*, *MAPK13*, and *SELPLG* in RTqPCR showed similar trends as recorded in RNA-Seq analysis. RT-qPCR, quantitative reverse transcription polymerase chain reaction; *CCL5*, C-C motif chemokine ligand 5; *TNFSF13*, TNF superfamily 13; *BLA-DQB*, major histocompatibility complex antigen class II; *CXCR2*, C-X-C motif chemokine receptor 2; *NFKBIA*, NF-kappa-B inhibitor alpha; *CD4*, cluster difference 4; *MAPK13*, mitogen-activated protein kinase 13; *SELPLG*, selectin P ligand.

Finally, we validated the RNA-seq data by conducting RT-qPCR for eight randomly selected genes (*MAPK13*, *CD4*, *SELPLG*, *CXCR2*, *BLA-DQB*, *CCL5*, *NFKBIA*, and *TNFSF13*). The results showed that all the genes had similar expression trends as detected in the RNA-seq. This consistency between RT-qPCR and RNA-seq revealed the reliability of our RNA-Seq data (Figure 6).

## DISCUSSION

Folate is known to be one of the most key nutrients having an essential role in the improvement of immunity and prevention of diseases for the pregnant mammals. However, in dairy cattle, external folate intake has been widely neglected especially during the perinatal period. The current research using RNA-seq analysis proved the importance of folic acid, a synthetic form of folate, in terms of immunity and health regulation in transition Holstein cows.

The periparturient period in mammals is known to be critical for fecundity and health. During the periparturient period, the preservation of health and the preparation for oncoming parturition is of supreme significance for dairy cows. Balanced nutrition is necessary for maintaining a functional immune system, while also for avoiding other causes of inflammation, such as tissue damage, digestive, metabolic disorders, and infectious diseases during the perinatal period [11]. In the current study, we tested the influence of orally administered coated folic acid in Holstein cows during the periparturient period and observed that the serum cytokines IL-17 and IFN-γ were up-regulated after low folic acid supplementation. IL-17 plays a key role in the host defenses against bacterial and fungal infections [12–15]. Our recent work found that the mutations in IL-17 might serve as biomarkers for resistance against mastitis in Holstein cattle as well as Sanhe cattle, a dual purpose breed of China [16]. Moreover, IFN-γ is important for immune regulation as well as for the promotion of T cell priming and antibodies production [17–19]. As during the periparturient period immunity is suppressed and dairy cows are prone to infection at this time [20], the elevated levels of IL17 and IFN-γ in response to low folic acid treatment suggest that dairy cows during the periparturient period should be fed with folic acid as matter of prophylaxis.

For more understanding, we conducted the transcriptomic study of PBLs for the three dairy cattle groups with high, low and none folic acid supplementation. Previous studies reported that many biological pathways play a key role by accelerating immune-relevant cells and provide a strong defense against any foreign pathogens [21]. The nuclear factor (NF)-kappa B signaling pathway, hematopoietic cell lineage, and TNF signaling pathways were reported to respond to *Staphylococcus aureus* (*S. aureus*) in mammary epithelial cells [22], and against porcine reproductive and respiratory syndrome virus post-vaccination in pregnant sows [23]. Moreover, studies also revealed that the toll like receptor signaling pathway plays a significant role in adaptive immunity [24,25]. Interestingly, we documented the above immunity associated pathways such as chemokine signaling pathway, TNF signaling, and NF-kappa B signaling pathway significantly (p<0.05) responded to high and low coated folic acid supplementation. Using microarrays, Ouattara et al [6] found that intra-muscular injection of vitamins B_9_ (folate) and B_12_ positively regulated the biological processes having fundamental importance in immune, anti-inflammation, cell adhesion and response to stress in hepatic and mammary gland gene expression profiles in lactating dairy cows. The antigen presentation and processing signaling, cytokine-cytokine receptor interaction, and *S. aureus* infection which are regarded as the key immunity associated biological pathways [23,26] were also reported in our low folic acid treated cows. Especially, linear regression analyses showed moderate relationships between the expression levels of differentially expressed genes (*CX3CR1* and *CCR3* in cytokine-cytokine receptor interaction pathway, as well as *BoLA-DQA5* in antigen presentation and processing signaling pathway and *S. aureus* infection pathway) and serum IL-17 (Supplementary file 8). Our study revealed that immunity linked biological function processes, i.e., immune response, defense response to bacterium, inflammatory function, chemokines, and cytokines mediated functions were dominantly regulated by orally administered folic acid (high, low). These findings suggest that coated folic acid oral supplementation provoke the immunity-related pathways and biological processes which may preserve the health and improve production performance of dairy cattle.

**Figure 7:**
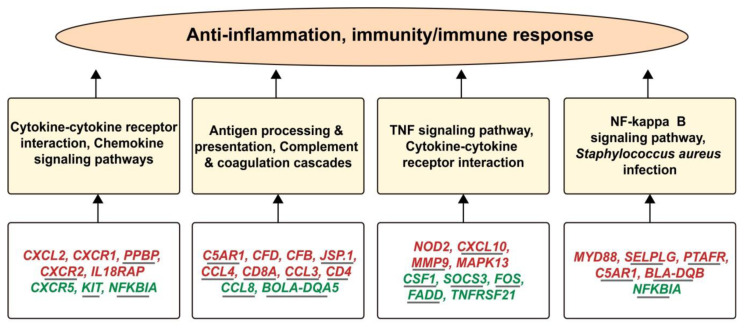
The interactive relationships among differentially expressed genes (lower panels), pathways (middle panels) and immunity response (upper panel) involved in the regulation of folic acid in perinatal Holstein cows. The genes in green represent down-regulated, while genes in red mean up-regulated. Additionally, the genes regulated in low and control folic acid comparison are underlined.

To control the related traits normally, a close correlation of genes with each other within the pathways is obligatory. When these genes are influenced by any external or internal factor, it will create a series of changes in the function of the body. In the current research (Figure 7), we proved that folic acid supplementation causes the down-regulation of *NFKBIA* and TNF alpha-induced protein 3 (*TNFAIP3*) which are involved in the negative mediation of NF-kappaB transcription factor activity. The suppressor of cytokine signaling 3 (*SOCS3*) was also significantly down-regulated which is involved in the negative regulation of cytokines that signal through the JAK/STAT pathway. Additionally, the *MYD88*, nucleotide-binding oligomerization domain containing 2 and *MAPK13* are the positive regulators of IL-6 were successfully up-regulated by the folate treatment. The folic acid supplementation also regulated many important genes associated with immune response such as MHC class II antigen (*BLA-DQB*), *TNFRSF1A*, *CXCL8*, *CXCL10*, *PTAFR*, *CXCR1*, *CXCR5*, and *CCL5*. Importantly, our previously published study [22] documented the up-regulation of the *CXCR1*, *NFKBIA*, *SOCS3*, Pim-1 proto-oncogene, serine/threonine kinase, dual specificity phosphatase 4, zinc finger CCCH-type containing 12A, and *NFKBIA* in mammary epithelial cells after infection by *S. aureus in vitro*, while these genes showed significantly downregulated status in folic acid treated cows. In addition, *NFKBIA* was up-regulated in response to Duck Tampusu virus infection [27]. Meanwhile, we also noticed several key up-regulated genes (*CD8A*, *CCL3*, *CD4*, *BLA-DQB*, etc.) which play important roles in the development of immunity (Supplementary file 7) were only detected in low folic acid treatment. Taking the up-regulated *CD4* gene as an example, which has an important function in the development of immunity, was down-regulated in mastitis cows [28]. Collectively, the data suggest that low folic acid supplementation (120 mg/500 kg cow/d) has key role in the mediation of immunity associated genes, which might be used as biomarkers for health regulation.

A few studies in humans reported that the dietary folic acid in a high dose for an extended period had an adverse effect on natural killer cell cytotoxicity [29]. Controversially, we did not report any such kind of change related to genes reported in our selected Chinese Holstein cows. However, we noticed a higher level of cytokines and chemokines regulating genes in the comparison of high vs control which may be harmful to the body. In addition, it is alluring to contemplate that this variability might be due to the inconsistency in the period of folic acid supplementation. Although our study profiled for the first time the importance of folic acid in immune regulation signaling during the bovine perinatal period through transcriptomic analysis, however, there are still limitations in our research which need to be addressed in future studies. Firstly, the remarkably regulated pathways and DEGs need to be tested through further *in-vitro* study. Secondly, we noticed that metabolic associated biological pathways were also significantly regulated by folic acid treatment; thus future research on the metabolic-related traits linked to folic acid supplementation is warranted.

## CONCLUSION

To our knowledge, this is the first transcriptomic analysis of bovine lymphocytes in response to oral supplementation of coated folic acid (high and low folic acid) during the periparturient transition period. We demonstrated altered expression of a subset of key genes and pathways that seem to be associated with immune system capacity induced by folic acid, which collectively prepares the dairy cow for the immune challenges related to the perinatal period. Additionally, our research opens a new gateway for future research in the health regulation of dairy cattle during the periparturient period with the supplementation of folic acid.

## Figures and Tables

**Table 1:** Different biological pathways in response to high, low or non-folic acid supplementation in transition dairy cows

Pathway	Genes hits	p-value
High vs control		
bta04060: Cytokine-cytokine receptor interaction	34	1.30E-06
bta04668: TNF signaling pathway	17	3.40E-04
bta05150: *Staphylococcus aureus* infection	12	4.00E-04
bta04062: Chemokine signaling pathway	22	1.70E-03
bta04640: Hematopoietic cell lineage	13	5.70E-03
bta03320: PPAR signaling pathway	11	8.20E-03
bta04610: Complement and coagulation cascades	11	1.20E-02
bta04514: Cell adhesion molecules	17	1.40E-02
bta04064: NF-kappa B signaling pathway	11	3.20E-02
bta05200: Pathways in cancer	32	4.50E-02
Low vs control		
bta04514: Cell adhesion molecules	21	4.20E-09
bta04668: TNF signaling pathway	15	1.00E-06
bta05150: *Staphylococcus aureus* infection	10	2.40E-05
bta04640: Hematopoietic cell lineage	11	1.70E-04
bta04610: Complement and coagulation cascades	8	3.60E-03
bta04060: Cytokine-cytokine receptor interaction	15	4.80E-03
bta04612: Antigen processing and presentation	7	1.50E-02
bta04620: Toll-like receptor signaling pathway	8	2.30E-02
bta04062: Chemokine signaling pathway	11	2.70E-02
High vs low		
bta04640: Hematopoietic cell lineage	14	4.60E-06
bta04514: Cell adhesion molecules	14	9.80E-04
bta04060: Cytokine-cytokine receptor interaction	18	1.20E-03
bta04668: TNF signaling pathway	9	1.90E-02
bta05202: Transcriptional misregulation in cancer	12	2.00E-02

TNF, tumor necrosis factor; PPAR, peroxisome proliferator-activated receptor; NF, nuclear factor.

## References

[1] Abe I, Shirato K, Hashizume Y (2013). Folate-deficiency induced cell-specific changes in the distribution of lymphocytes and granulocytes in rats. Environ Health Prev Med.

[2] Lardinois CC, Mills RC, Elyehjem CA, Hart EB (1944). Rumen synthesis of the vitamin B complex as influenced by ration composition. J Dairy Sci.

[3] Aleri WJ, Hine CB, Pyman FM (2016). Periparturient immunosuppression and strategies to improve dairy cow health during the periparturient period. Res Vet Sci.

[4] Duplessis MS, Mann S, Nydam VD, Girard LC, Pellerin D, Overton RT (2015). Short communication: Folates and vitamin B12 in colostrum and milk from dairy cows fed different energy levels during the dry period. J Dairy Sci.

[5] Oshlack A, Robinson MD, Young MD (2010). From RNA-seq reads to differential expression results. Genome Biol.

[6] Ouattara B, Bissonnette N, Duplessis M, Girard CL (2016). Supplements of vitamins B_9_ and B_12_ affect hepatic and mammary gland gene expression profiles in lactating dairy cows. BMC Genomics.

[7] Duplessis M, Lapierre H, Pellerin D, Laforest JP, Girard CL (2017). Effects of intramuscular injections of folic acid, vitamin B_12_, or both, on lactational performance and energy status of multiparous dairy cows. J Dairy Sci.

[8] Graulet B, Matte JJ, Desrochers A, Doepel L, Palin MF, Girard CL (2007). Effects of dietary supplements of folic acid and vitamin B_12_ on metabolism of dairy cows in early lactation. J Dairy Sci.

[9] Trapnell C, Pachter L, Salzberg SL (2009). TopHat: discovering splice junctions with RNA-Seq. Bioinformatics.

[10] Trapnell C, Roberts A, Goff L (2012). Differential gene and transcript expression analysis of RNA-seq experiments with TopHat and Cufflinks. Nat Protoc.

[11] Bertoni G, Minuti A, Trevisi E (2015). Immune system, inflammation and nutrition in dairy cattle. Anim Prod Sci.

[12] Yoichiro I, Harumichi I, Shinobu S, Susumu N (2011). Functional specialization of interleukin-17 family members. Immunity.

[13] Khader SA, Bell GK, Pearl JE (2007). IL-23 and IL-17 in the establishment of protective pulmonary CD4+ T cell responses after vaccination and during *Mycobacterium tuberculosis* challenge. Nat Immunol.

[14] Park H, Li Z, Yang OX (2005). A distinct lineage of CD4 T cells regulates tissue inflammation by producing interleukin 17. Nat Immunol.

[15] Happel KI, Zheng M, Young E (2003). Roles of toll-like receptor 4 and IL-23 in IL-17 expression in response to *Klebsiella pneumoniae* infection. J Immunol.

[16] Usman T, Wang Y, Liu C (2017). Novel SNPs in *IL-17F* and *IL-17A* genes associated with somatic cell count in Chinese Holstein and Inner-Mongolia Sanhe cattle. J Anim Sci Biotechnol.

[17] Larkin J, Ahmed CM, Wilson TD, Johnson HM (2013). Regulation of interferon gamma signaling by suppressors of cytokine signaling and regulatory T cells. Front Immunol.

[18] Ayers M, Lunceford J, Nebozhyn M, McClanahan Terrill K (2017). IFN-γ–related mRNA profile predicts clinical response to PD-1 blockade. J Clin Invest.

[19] Schroder K, Hertzog PJ, Ravasi T, Hume DA (2004). Interferon-gamma: an overview of signals, mechanisms and functions. J Leukoc Biol.

[20] Mulligan FJ, Doherty ML (2008). Production diseases of the transition cow. Vet J.

[21] He Y, Song M, Zhang Y (2016). Whole-genome regulation analysis of histone H3 lysin 27 trimethylation in subclinical mastitis cows infected by *Staphylococcus aureus*. BMC Genomics.

[22] Fang L, Hou Y, An J (2016). Genome-wide transcriptional and post-transcriptional regulation of innate immune and defense responses of bovine mammary gland to *Staphylococcus aureus*. Front Cell Infect Microbiol.

[23] Yang T, Zhang F, Zhai L (2018). Transcriptome of porcine PBMCs over two generations reveals key genes and pathways associated with variable antibody responses post PRRSV vaccination. Sci Rep.

[24] Islam MA, Große-Brinkhaus C, Pröll MJ (2016). Deciphering transcriptome profiles of peripheral blood mononuclear cells in response to PRRSV vaccination in pigs. BMC Genomics.

[25] Schoenborn JR, Wilson CB (2007). Regulation of interferon-gamma during innate and adaptive immune responses. Adv Immunol.

[26] Kosciuczuk ME, Paweł L, Justyna J (2017). Transcriptome profiling of *Staphylococci* infected cow mammary gland parenchyma. BMC Vet Res.

[27] Yu G, Lin Y, Tang Y, Diao Y (2018). Comparative transcriptomic analysis of immune-related gene expression in duck embryo fibroblasts following duck Tembusu virus infection. Int J Mol Sci.

[28] Wang XS, Zhang Y, He YH (2013). Aberrant promoter methylation of the CD4 gene in peripheral blood cells of mastitic dairy cows. Genet Mol Res.

[29] Clovis P, Juliano FB, Maylla RL (2017). A daily dose of 5 mg folic acid for 90 days is associated with increased serum unmetabolized folic acid and reduced natural killer cell cytotoxicity in healthy Brazilian adults. J Nutr.

