# Supplementary material for: Folic acid supplementation regulates key immunity-associated genes and pathways during the periparturient period in dairy cows
Source: Asian-Australas J Anim Sci. 2019 Apr 15;33(9):1507–19. doi: 10.5713/ajas.18.0852 (PMC7468170; doi:10.5713/ajas.18.0852)
Supplement: Supplementary file 1 [file ajas-18-0852-suppl.pdf]

Supplementary file 1: List of primers used for the validation of RNA-seq results

**Gene name**	Sequence
CCL5	Forward: CTTTATCACCAGGAAGAAGCG<br>Reverse: GGACAAGAGCGAGAAGCAA
NFKBIA	F:CACTAACCAGCCAGAAATCG<br>R: TGACACCAGGTCGGGATT
TNFSF13	F:TCTCGTTCCCATTAACATTAC<br>R: AGCAGATAAACTCCAGCATC
BLA-DQB	F:TGCTCTTCTATAATGCGTGCCT<br>R:ACGGGAAATGACTTGGTGACTG
MAPK13	F: TGCTGACAGGGAAGACGC<br>R: GGCTTTGTGGCAGGGACT
SELPLG	F:CGCTACCGTTTCCTTCTACG<br>R: TCTGCGAGCACCTCAATC
CXCR2	F: TATGCCCTGGTCTTCTTGC<br>R:CAACCCTTTGCCTTGGAG
CD4	F:GGCAACCCACTCCAGTAC<br>R: GAGGCTGAAATAGACATTGTTA
GAPDH	F: GGTGCTGAGTATGTGGTGGA<br>R: GGCATTGCTGACAATCTTGA

Supplementary file 2. Pathways

High folic acid treated group regulated pathways

Term	Count	P- value	Genes
bta04060:Cytokine-cytokine receptor interaction	34	1.29E-06	ENSBTAG00000018424, ENSBTAG00000014113, ENSBTAG00000005273, ENSBTAG00000000283, ENSBTAG00000026753, ENSBTAG00000008299, ENSBTAG00000013586, ENSBTAG00000019428, ENSBTAG00000013334, ENSBTAG00000011909, ENSBTAG00000032425, ENSBTAG00000001034, ENSBTAG00000000130, ENSBTAG00000005359, ENSBTAG00000004211, ENSBTAG00000016345, ENSBTAG00000015133, ENSBTAG00000019716, ENSBTAG00000046320, ENSBTAG00000002923, ENSBTAG00000033748, ENSBTAG00000007191, ENSBTAG00000019338, ENSBTAG00000038042, ENSBTAG00000013205, ENSBTAG00000003289, ENSBTAG00000012544, ENSBTAG00000020054, ENSBTAG00000001725, ENSBTAG00000012771, ENSBTAG00000006519, ENSBTAG00000012899, ENSBTAG00000006343, ENSBTAG00000013555
bta04380:Osteoclast differentiation	21	5.78E-05	ENSBTAG00000007213, ENSBTAG00000021358, ENSBTAG00000002103, ENSBTAG00000008441, ENSBTAG00000005273, ENSBTAG00000000283, ENSBTAG00000007275, ENSBTAG00000001333, ENSBTAG00000002922, ENSBTAG00000006679, ENSBTAG00000005841, ENSBTAG00000012544, ENSBTAG00000005359, ENSBTAG00000012771, ENSBTAG00000004211, ENSBTAG00000007867, ENSBTAG00000010007, ENSBTAG00000016683, ENSBTAG00000012899, ENSBTAG00000007338, ENSBTAG00000004386
bta00480:Glutathione metabolism	12	1.75E-04	ENSBTAG00000007115, ENSBTAG00000005989, ENSBTAG00000020527, ENSBTAG00000013527, ENSBTAG00000015571, ENSBTAG00000018281, ENSBTAG00000019512, ENSBTAG00000008541, ENSBTAG00000004288, ENSBTAG00000043553, ENSBTAG00000046900, ENSBTAG00000021779
bta04668:TNF signaling pathway	17	3.43E-04	ENSBTAG00000008441, ENSBTAG00000037539, ENSBTAG00000000283, ENSBTAG00000020936, ENSBTAG00000015727, ENSBTAG00000007191, ENSBTAG00000001034, ENSBTAG00000011600, ENSBTAG00000027513, ENSBTAG00000020676, ENSBTAG00000009493, ENSBTAG00000001725, ENSBTAG00000004211, ENSBTAG00000016683, ENSBTAG00000010007, ENSBTAG00000000436, ENSBTAG00000010303

bta05150:Staphylococcus aureus infection	12	3.96E-04	ENSBTAG00000019588, ENSBTAG00000031851, ENSBTAG00000038128, ENSBTAG00000021077, ENSBTAG00000027051, ENSBTAG00000021647, ENSBTAG00000020872, ENSBTAG00000046158, ENSBTAG00000010303, ENSBTAG00000048122, ENSBTAG00000011196, ENSBTAG00000007153
bta01230:Biosynthesis of amino acids	13	8.22E-04	ENSBTAG00000008945, ENSBTAG00000001601, ENSBTAG00000013099, ENSBTAG00000002980, ENSBTAG00000003758, ENSBTAG00000013631, ENSBTAG00000012927, ENSBTAG00000010336, ENSBTAG00000020527, ENSBTAG00000047806, ENSBTAG00000020747, ENSBTAG00000012403, ENSBTAG00000031814
bta01130:Biosynthesis of antibiotics	24	1.31E-03	ENSBTAG00000008945, ENSBTAG00000010206, ENSBTAG00000001601, ENSBTAG00000018936, ENSBTAG00000013527, ENSBTAG00000003196, ENSBTAG00000013099, ENSBTAG00000003948, ENSBTAG00000010801, ENSBTAG00000003758, ENSBTAG00000012927, ENSBTAG00000010336, ENSBTAG00000012961, ENSBTAG00000008683, ENSBTAG00000047806, ENSBTAG00000020747, ENSBTAG00000020527, ENSBTAG00000012403, ENSBTAG00000016211, ENSBTAG00000031814, ENSBTAG00000019512, ENSBTAG00000004092, ENSBTAG00000019315, ENSBTAG00000015821
bta04062:Chemokine signaling pathway	22	1.71E-03	ENSBTAG00000014113, ENSBTAG00000002767, ENSBTAG00000035587, ENSBTAG00000026753, ENSBTAG00000046320, ENSBTAG00000002923, ENSBTAG00000009812, ENSBTAG00000013586, ENSBTAG00000019428, ENSBTAG00000007932, ENSBTAG00000032425, ENSBTAG00000017839, ENSBTAG00000007191, ENSBTAG00000007193, ENSBTAG00000038042, ENSBTAG00000003289, ENSBTAG00000027513, ENSBTAG00000001725, ENSBTAG00000007867, ENSBTAG00000016683, ENSBTAG00000015133, ENSBTAG00000019716
bta05321:Inflammatory bowel disease (IBD)	12	1.75E-03	ENSBTAG00000019588, ENSBTAG00000014199, ENSBTAG00000038128, ENSBTAG00000021077, ENSBTAG00000001034, ENSBTAG00000012544, ENSBTAG00000005359, ENSBTAG00000007867, ENSBTAG00000020936, ENSBTAG00000033748, ENSBTAG00000012899, ENSBTAG00000012599

bta05164:Influenza A	21	1.75E-03	ENSBTAG00000040569, ENSBTAG00000019588, ENSBTAG00000008441, ENSBTAG00000021077, ENSBTAG00000037527, ENSBTAG00000003948, ENSBTAG00000007191, ENSBTAG00000038128, ENSBTAG00000039861, ENSBTAG00000025441, ENSBTAG00000000563, ENSBTAG00000012544, ENSBTAG00000001725, ENSBTAG00000004211, ENSBTAG00000007867, ENSBTAG00000016683, ENSBTAG00000012899, ENSBTAG00000010007, ENSBTAG00000010303, ENSBTAG00000020649, ENSBTAG00000019716
bta05323:Rheumatoid arthritis	14	2.53E-03	ENSBTAG00000019588, ENSBTAG00000021077, ENSBTAG00000000283, ENSBTAG00000009812, ENSBTAG00000012117, ENSBTAG00000019218, ENSBTAG00000007191, ENSBTAG00000038128, ENSBTAG00000000130, ENSBTAG00000003289, ENSBTAG00000005359, ENSBTAG00000010303, ENSBTAG00000013170, ENSBTAG00000019716
bta05144:Malaria	10	2.62E-03	ENSBTAG00000037539, ENSBTAG00000000563, ENSBTAG00000017866, ENSBTAG00000005359, ENSBTAG00000026417, ENSBTAG00000010303, ENSBTAG00000026418, ENSBTAG00000039325, ENSBTAG00000010830, ENSBTAG00000019716
bta00030:Pentose phosphate pathway	7	3.39E-03	ENSBTAG00000047806, ENSBTAG00000013527, ENSBTAG00000019512, ENSBTAG00000013099, ENSBTAG00000003758, ENSBTAG00000012927, ENSBTAG00000010336
bta05143:African trypanosomiasis	8	4.14E-03	ENSBTAG00000034848, ENSBTAG00000037539, ENSBTAG00000000563, ENSBTAG00000020602, ENSBTAG00000009789, ENSBTAG00000026417, ENSBTAG00000010303, ENSBTAG00000026418
bta04640:Hematopoietic cell lineage	13	5.42E-03	ENSBTAG00000005273, ENSBTAG00000032122, ENSBTAG00000000283, ENSBTAG00000024960, ENSBTAG00000013334, ENSBTAG00000039046, ENSBTAG00000017866, ENSBTAG00000012771, ENSBTAG00000009421, ENSBTAG00000002302, ENSBTAG00000014764, ENSBTAG00000006343, ENSBTAG00000004608
bta03320:PPAR signaling pathway	11	5.66E-03	ENSBTAG00000017851, ENSBTAG00000004547, ENSBTAG00000012855, ENSBTAG00000017866, ENSBTAG00000001333, ENSBTAG00000032436, ENSBTAG00000010681, ENSBTAG00000019708, ENSBTAG00000012205, ENSBTAG00000013489, ENSBTAG00000017542

bta05145:Toxoplasmosis	15	8.16E-03	ENSBTAG00000019588, ENSBTAG00000021077, ENSBTAG00000020319, ENSBTAG00000038128, ENSBTAG00000025441, ENSBTAG00000000563, ENSBTAG00000005359, ENSBTAG00000012544, ENSBTAG00000007867, ENSBTAG00000004211, ENSBTAG00000016683, ENSBTAG00000012899, ENSBTAG00000010007, ENSBTAG00000020649, ENSBTAG00000004386
bta04610:Complement and coagulation cascades	11	8.39E-03	ENSBTAG00000012265, ENSBTAG00000002758, ENSBTAG00000020872, ENSBTAG00000002302, ENSBTAG00000017722, ENSBTAG00000007268, ENSBTAG00000046158, ENSBTAG00000010520, ENSBTAG00000048122, ENSBTAG00000011196, ENSBTAG00000007153
bta05142:Chagas disease (American trypanosomiasis)	14	1.19E-02	ENSBTAG00000012599, ENSBTAG00000011196, ENSBTAG00000007191, ENSBTAG00000000563, ENSBTAG00000003289, ENSBTAG00000012544, ENSBTAG00000009789, ENSBTAG00000005359, ENSBTAG00000004211, ENSBTAG00000012899, ENSBTAG00000010007, ENSBTAG00000016683, ENSBTAG00000019716, ENSBTAG00000007153
bta04514:Cell adhesion molecules (CAMs)	17	1.33E-02	ENSBTAG00000019588, ENSBTAG00000021077, ENSBTAG00000015318, ENSBTAG00000037539, ENSBTAG00000020116, ENSBTAG00000000603, ENSBTAG00000014906, ENSBTAG00000011543, ENSBTAG00000000977, ENSBTAG00000000095, ENSBTAG00000017846, ENSBTAG00000038128, ENSBTAG00000031851, ENSBTAG00000015742, ENSBTAG00000003176, ENSBTAG00000010303, ENSBTAG00000013170
bta05152:Tuberculosis	19	1.37E-02	ENSBTAG00000019588, ENSBTAG00000021077, ENSBTAG00000017135, ENSBTAG00000012185, ENSBTAG00000020936, ENSBTAG00000012117, ENSBTAG00000019218, ENSBTAG00000008507, ENSBTAG00000038128, ENSBTAG00000006572, ENSBTAG00000000563, ENSBTAG00000012544, ENSBTAG00000005359, ENSBTAG00000004211, ENSBTAG00000007867, ENSBTAG00000024503, ENSBTAG00000012899, ENSBTAG00000010007, ENSBTAG00000020649
bta05140:Leishmaniasis	10	1.80E-02	ENSBTAG00000019588, ENSBTAG00000038128, ENSBTAG00000021077, ENSBTAG00000000563, ENSBTAG00000012544, ENSBTAG00000005359, ENSBTAG00000007867, ENSBTAG00000016683, ENSBTAG00000010007, ENSBTAG00000012899

bta04145:Phagosome	17	1.96E-02	ENSBTAG00000014269, ENSBTAG00000019588, ENSBTAG00000023730, ENSBTAG00000021013, ENSBTAG00000021077, ENSBTAG00000004547, ENSBTAG00000017135, ENSBTAG00000020116, ENSBTAG00000021647, ENSBTAG00000001489, ENSBTAG00000002177, ENSBTAG00000039992, ENSBTAG00000012117, ENSBTAG00000019218, ENSBTAG00000038128, ENSBTAG00000017866, ENSBTAG00000002885
bta01200:Carbon metabolism	13	1.98E-02	ENSBTAG00000008945, ENSBTAG00000001601, ENSBTAG00000013527, ENSBTAG00000013099, ENSBTAG00000002980, ENSBTAG00000003758, ENSBTAG00000012927, ENSBTAG00000010336, ENSBTAG00000018201, ENSBTAG00000020527, ENSBTAG00000047806, ENSBTAG00000031814, ENSBTAG00000019512
bta05168:Herpes simplex infection	19	2.15E-02	ENSBTAG00000019588, ENSBTAG00000008441, ENSBTAG00000021077, ENSBTAG00000015318, ENSBTAG00000007881, ENSBTAG00000020116, ENSBTAG00000037527, ENSBTAG00000001066, ENSBTAG00000007191, ENSBTAG00000038128, ENSBTAG00000039861, ENSBTAG00000000693, ENSBTAG00000000563, ENSBTAG00000012544, ENSBTAG00000004211, ENSBTAG00000007867, ENSBTAG00000016683, ENSBTAG00000012899, ENSBTAG00000015815
bta04142:Lysosome	14	2.55E-02	ENSBTAG00000017135, ENSBTAG00000011931, ENSBTAG00000021829, ENSBTAG00000012442, ENSBTAG00000008849, ENSBTAG00000012117, ENSBTAG00000010992, ENSBTAG00000019218, ENSBTAG00000015520, ENSBTAG00000000133, ENSBTAG00000004436, ENSBTAG00000009506, ENSBTAG00000016021, ENSBTAG00000011100
bta04064:NF-kappa B signaling pathway	11	3.22E-02	ENSBTAG00000021358, ENSBTAG00000002103, ENSBTAG00000005273, ENSBTAG00000037539, ENSBTAG00000000563, ENSBTAG00000004211, ENSBTAG00000016683, ENSBTAG00000000436, ENSBTAG00000010303, ENSBTAG00000019634, ENSBTAG00000019716

			ENSBTAG00000006156, ENSBTAG00000031358,
			ENSBTAG00000030333, ENSBTAG00000003196,
			ENSBTAG00000017592, ENSBTAG00000010801,
			ENSBTAG00000008507, ENSBTAG00000037726,
			ENSBTAG00000032954, ENSBTAG00000007681,
			ENSBTAG00000002346, ENSBTAG00000012519,
			ENSBTAG00000020527, ENSBTAG00000032996,
			ENSBTAG00000005586, ENSBTAG00000046900,
			ENSBTAG00000008945, ENSBTAG00000009737,
			ENSBTAG00000001154, ENSBTAG00000046750,
			ENSBTAG00000020319, ENSBTAG00000010336,
			ENSBTAG00000018186, ENSBTAG00000008683,
			ENSBTAG00000011278, ENSBTAG00000020747,
			ENSBTAG00000016211, ENSBTAG00000019708,
			ENSBTAG00000010033, ENSBTAG00000016021,
			ENSBTAG00000004092, ENSBTAG00000020225,
			ENSBTAG00000009603, ENSBTAG00000006136,
			ENSBTAG00000046152, ENSBTAG00000016206,
			ENSBTAG00000013527, ENSBTAG00000020602,
			ENSBTAG00000019272, ENSBTAG00000013099,
			ENSBTAG00000003948, ENSBTAG00000002980,
			ENSBTAG00000000087, ENSBTAG00000019218,
			ENSBTAG00000003901, ENSBTAG00000013093,
			ENSBTAG00000018101, ENSBTAG00000016869,
			ENSBTAG00000011558, ENSBTAG00000004436,
			ENSBTAG00000018201, ENSBTAG00000011990,
			ENSBTAG00000005989, ENSBTAG00000017676,
			ENSBTAG00000018744, ENSBTAG00000008951,
bta01100:Metabolic pathways	86	3.28E-02	ENSBTAG00000000033, ENSBTAG00000047806,
			ENSBTAG00000007684, ENSBTAG00000014595,
			ENSBTAG00000002742, ENSBTAG00000016758,
bta03430:Mismatch repair	5	3.80E-02	ENSBTAG00000016869
			ENSBTAG00000011150, ENSBTAG00000000563,
			ENSBTAG00000000347, ENSBTAG00000027513,
			ENSBTAG00000003289, ENSBTAG00000015278,
			ENSBTAG00000012544, ENSBTAG00000010007,
bta05132:Salmonella infection	10	4.40E-02	ENSBTAG00000012899, ENSBTAG00000019716

bta05200:Pathways in cancer	32	4.47E-02	ENSBTAG00000002103, ENSBTAG00000007684, ENSBTAG00000020957, ENSBTAG00000021798, ENSBTAG00000012599, ENSBTAG00000013334, ENSBTAG00000009498, ENSBTAG00000005359, ENSBTAG00000009789, ENSBTAG00000008300, ENSBTAG00000006998, ENSBTAG00000008409, ENSBTAG00000021527, ENSBTAG00000008006, ENSBTAG00000044023, ENSBTAG00000019716, ENSBTAG00000002767, ENSBTAG00000002742, ENSBTAG00000013573, ENSBTAG00000001333, ENSBTAG00000020194, ENSBTAG00000006679, ENSBTAG00000017851, ENSBTAG00000019354, ENSBTAG00000016758, ENSBTAG00000012771, ENSBTAG00000020676, ENSBTAG00000007867, ENSBTAG00000016683, ENSBTAG00000038990, ENSBTAG00000003791, ENSBTAG00000017542
bta04920:Adipocytokine signaling pathway	9	4.61E-02	ENSBTAG00000017851, ENSBTAG00000008441, ENSBTAG00000017866, ENSBTAG00000007166, ENSBTAG00000013705, ENSBTAG00000004211, ENSBTAG00000016683, ENSBTAG00000019708, ENSBTAG00000012205
bta00590:Arachidonic acid metabolism	9	5.29E-02	ENSBTAG00000011990, ENSBTAG00000009737, ENSBTAG00000018281, ENSBTAG00000010033, ENSBTAG00000013298, ENSBTAG00000020319, ENSBTAG00000043553, ENSBTAG00000046900, ENSBTAG00000020225
bta00270:Cysteine and methionine metabolism	6	6.11E-02	ENSBTAG00000021143, ENSBTAG00000008683, ENSBTAG00000008945, ENSBTAG00000031814, ENSBTAG00000003901, ENSBTAG00000018101
bta05202:Transcriptional misregulation in cancer	16	6.74E-02	ENSBTAG00000018423, ENSBTAG00000005757, ENSBTAG00000005952, ENSBTAG00000039813, ENSBTAG00000001333, ENSBTAG00000017851, ENSBTAG00000019354, ENSBTAG00000008966, ENSBTAG00000020676, ENSBTAG00000012771, ENSBTAG00000016345, ENSBTAG00000015133, ENSBTAG00000006343, ENSBTAG00000008409, ENSBTAG00000021527, ENSBTAG00000019716
bta04621:NOD-like receptor signaling pathway	7	7.17E-02	ENSBTAG00000007191, ENSBTAG00000006572, ENSBTAG00000020936, ENSBTAG00000016683, ENSBTAG00000010007, ENSBTAG00000000436, ENSBTAG00000019716
bta05160:Hepatitis C	13	7.29E-02	ENSBTAG00000017851, ENSBTAG00000014269, ENSBTAG00000008441, ENSBTAG00000039861, ENSBTAG00000007881, ENSBTAG00000037527, ENSBTAG00000010681, ENSBTAG00000004211, ENSBTAG00000007867, ENSBTAG00000016683, ENSBTAG00000010007, ENSBTAG00000031231, ENSBTAG00000019716

bta04670:Leukocyte transendothelial migration	12	7.31E-02	ENSBTAG00000002103, ENSBTAG00000019627, ENSBTAG00000002411, ENSBTAG00000037539, ENSBTAG00000000603, ENSBTAG00000011473, ENSBTAG00000020676, ENSBTAG00000003176, ENSBTAG00000010007, ENSBTAG00000010303, ENSBTAG00000017846, ENSBTAG00000005557
bta00220:Arginine biosynthesis	4	8.89E-02	ENSBTAG00000020747, ENSBTAG00000012403, ENSBTAG00000002980, ENSBTAG00000013631
bta04918:Thyroid hormone synthesis	8	9.38E-02	ENSBTAG00000013245, ENSBTAG00000019354, ENSBTAG00000007115, ENSBTAG00000015392, ENSBTAG00000009789, ENSBTAG00000018281, ENSBTAG00000020455, ENSBTAG00000043553

Low folic acid treated group regulated pathways

Term	Count	P- value	Genes
bta04514:Cell adhesion molecules (CAMs)	21	4.19E-09	ENSBTAG00000019588, ENSBTAG00000002069, ENSBTAG00000021141, ENSBTAG00000037539, ENSBTAG00000019097, ENSBTAG00000020116, ENSBTAG00000000603, ENSBTAG00000015991, ENSBTAG00000007103, ENSBTAG00000000095, ENSBTAG00000007235, ENSBTAG00000017846, ENSBTAG00000019734, ENSBTAG00000008956, ENSBTAG00000026326, ENSBTAG00000038128, ENSBTAG00000031851, ENSBTAG00000015742, ENSBTAG00000013462, ENSBTAG00000010303, ENSBTAG00000003255
bta04668:TNF signaling pathway	15	1.00E-06	ENSBTAG00000018274, ENSBTAG00000008441, ENSBTAG00000037539, ENSBTAG00000000283, ENSBTAG00000006143, ENSBTAG00000007191, ENSBTAG00000012046, ENSBTAG00000011600, ENSBTAG00000004322, ENSBTAG00000020676, ENSBTAG00000009493, ENSBTAG00000001725, ENSBTAG00000016683, ENSBTAG00000000436, ENSBTAG00000010303
bta05150:Staphylococcus aureus infection	10	2.42E-05	ENSBTAG00000019588, ENSBTAG00000031851, ENSBTAG00000038128, ENSBTAG00000027051, ENSBTAG00000007103, ENSBTAG00000020872, ENSBTAG00000011193, ENSBTAG00000010303, ENSBTAG00000011196, ENSBTAG00000007153
bta04640:Hematopoietic cell lineage	11	1.68E-04	ENSBTAG00000039046, ENSBTAG00000021141, ENSBTAG00000000283, ENSBTAG00000038496, ENSBTAG00000024960, ENSBTAG00000002699, ENSBTAG00000002302, ENSBTAG00000007558, ENSBTAG00000003255, ENSBTAG00000008956, ENSBTAG00000004608

bta05323:Rheumatoid arthritis	10	9.83E-04	ENSBTAG00000007191, ENSBTAG00000019588, ENSBTAG00000038128, ENSBTAG00000000130, ENSBTAG00000000283, ENSBTAG00000004322, ENSBTAG00000005359, ENSBTAG00000007103, ENSBTAG00000010303, ENSBTAG00000025250
bta05142:Chagas disease (American trypanosomiasis)	10	3.50E-03	ENSBTAG00000018317, ENSBTAG00000007191, ENSBTAG00000018274, ENSBTAG00000004322, ENSBTAG00000005359, ENSBTAG00000016683, ENSBTAG00000011193, ENSBTAG00000025250, ENSBTAG00000011196, ENSBTAG00000007153
bta04610:Complement and coagulation cascades	8	3.60E-03	ENSBTAG00000038496, ENSBTAG00000020872, ENSBTAG00000002302, ENSBTAG00000016267, ENSBTAG00000011193, ENSBTAG00000010520, ENSBTAG00000011196, ENSBTAG00000007153
bta04060:Cytokine-cytokine receptor interaction	15	4.83E-03	ENSBTAG00000014113, ENSBTAG00000000283, ENSBTAG00000007558, ENSBTAG00000002923, ENSBTAG00000001338, ENSBTAG00000011909, ENSBTAG00000032425, ENSBTAG00000007191, ENSBTAG00000000130, ENSBTAG00000038042, ENSBTAG00000005359, ENSBTAG00000002699, ENSBTAG00000001725, ENSBTAG00000025250, ENSBTAG00000025257
bta05169:Epstein-Barr virus infection	13	5.53E-03	ENSBTAG00000014223, ENSBTAG00000002069, ENSBTAG00000020116, ENSBTAG00000038496, ENSBTAG00000007103, ENSBTAG00000026326, ENSBTAG00000013705, ENSBTAG00000016683, ENSBTAG00000010303, ENSBTAG00000000436, ENSBTAG00000008353, ENSBTAG00000008409, ENSBTAG00000013569
bta05020:Prion diseases	5	1.01E-02	ENSBTAG00000007191, ENSBTAG00000011193, ENSBTAG00000010520, ENSBTAG00000011196, ENSBTAG00000007153
bta00590:Arachidonic acid metabolism	7	1.34E-02	ENSBTAG00000011990, ENSBTAG00000009737, ENSBTAG00000016415, ENSBTAG00000010033, ENSBTAG00000020319, ENSBTAG00000046900, ENSBTAG00000006608
bta00480:Glutathione metabolism	6	1.46E-02	ENSBTAG00000005989, ENSBTAG00000001842, ENSBTAG00000010265, ENSBTAG00000004288, ENSBTAG00000046900, ENSBTAG00000006608
bta04612:Antigen processing and presentation	7	1.52E-02	ENSBTAG00000019588, ENSBTAG00000002069, ENSBTAG00000038128, ENSBTAG00000021141, ENSBTAG00000020116, ENSBTAG00000003255, ENSBTAG00000008956
bta05219:Bladder cancer	5	2.18E-02	ENSBTAG00000015991, ENSBTAG00000020676, ENSBTAG00000011820, ENSBTAG00000008353, ENSBTAG00000008409

bta04620:Toll-like receptor signaling pathway	8	2.28E-02	ENSBTAG00000007191, ENSBTAG00000018274, ENSBTAG00000011600, ENSBTAG00000004322, ENSBTAG00000001725, ENSBTAG00000016683, ENSBTAG00000025250, ENSBTAG00000025257
bta04062:Chemokine signaling pathway	11	2.74E-02	ENSBTAG00000007191, ENSBTAG00000014113, ENSBTAG00000038042, ENSBTAG00000001725, ENSBTAG00000002923, ENSBTAG00000016683, ENSBTAG00000025250, ENSBTAG00000009938, ENSBTAG00000025257, ENSBTAG00000001338, ENSBTAG00000032425
bta04380:Osteoclast differentiation	9	2.78E-02	ENSBTAG00000008441, ENSBTAG00000012046, ENSBTAG00000000283, ENSBTAG00000008182, ENSBTAG00000004322, ENSBTAG00000005359, ENSBTAG00000001333, ENSBTAG00000016683, ENSBTAG00000004386
bta05166:HTLV-I infection	14	2.93E-02	ENSBTAG00000019588, ENSBTAG00000014223, ENSBTAG00000038128, ENSBTAG00000002069, ENSBTAG00000012567, ENSBTAG00000037539, ENSBTAG00000020116, ENSBTAG00000005359, ENSBTAG00000004322, ENSBTAG00000007103, ENSBTAG00000016683, ENSBTAG00000010303, ENSBTAG00000008353, ENSBTAG00000008409
bta05416:Viral myocarditis	6	3.76E-02	ENSBTAG00000019588, ENSBTAG00000002069, ENSBTAG00000038128, ENSBTAG00000020116, ENSBTAG00000007103, ENSBTAG00000010303
bta05144:Malaria	5	5.35E-02	ENSBTAG00000037539, ENSBTAG00000005359, ENSBTAG00000007103, ENSBTAG00000010303, ENSBTAG00000004917
bta04660:T cell receptor signaling pathway	7	7.06E-02	ENSBTAG00000021141, ENSBTAG00000011600, ENSBTAG00000004322, ENSBTAG00000013705, ENSBTAG00000016683, ENSBTAG00000003255, ENSBTAG00000008956
bta00770:Pantothenate and CoA biosynthesis	3	8.31E-02	ENSBTAG00000012279, ENSBTAG00000021830, ENSBTAG00000016357
bta04010:MAPK signaling pathway	12	8.60E-02	ENSBTAG00000025462, ENSBTAG00000017512, ENSBTAG00000044208, ENSBTAG00000011600, ENSBTAG00000003033, ENSBTAG00000004322, ENSBTAG00000005359, ENSBTAG00000016442, ENSBTAG00000020194, ENSBTAG00000020283, ENSBTAG00000013761, ENSBTAG00000008409

high vs. Low folic acid treated group regulated pathways

**Term**	Count	P- value	Genes

**bta04640:Hematopoietic cell lineage**	14	4.64E-06	ENSBTAG00000000273, ENSBTAG00000021141, ENSBTAG00000015032, ENSBTAG00000038496, ENSBTAG00000007558, ENSBTAG00000008956, ENSBTAG00000011887, ENSBTAG00000039046, ENSBTAG00000039366, ENSBTAG00000017866, ENSBTAG00000011359, ENSBTAG00000012771, ENSBTAG00000014764, ENSBTAG00000006343
**bta04514:Cell adhesion molecules (CAMs)**	14	9.85E-04	ENSBTAG00000002069, ENSBTAG00000021141, ENSBTAG00000015318, ENSBTAG00000019097, ENSBTAG00000015991, ENSBTAG00000014906, ENSBTAG00000019734, ENSBTAG00000008956, ENSBTAG00000011887, ENSBTAG00000019432, ENSBTAG00000038128, ENSBTAG00000040432, ENSBTAG00000013462, ENSBTAG00000003176
**bta04060:Cytokine-cytokine receptor interaction**	18	1.16E-03	ENSBTAG00000000273, ENSBTAG00000046320, ENSBTAG00000002923, ENSBTAG00000007558, ENSBTAG00000033748, ENSBTAG00000019428, ENSBTAG00000007191, ENSBTAG00000017664, ENSBTAG00000018200, ENSBTAG00000019338, ENSBTAG00000013205, ENSBTAG00000020054, ENSBTAG00000012771, ENSBTAG00000004211, ENSBTAG00000005077, ENSBTAG00000025257, ENSBTAG00000006343, ENSBTAG00000013555

**bta01100:Metabolic pathways**	56	3.55E-03	ENSBTAG00000031358, ENSBTAG00000003196, ENSBTAG00000017592, ENSBTAG00000008507, ENSBTAG00000037726, ENSBTAG00000007681, ENSBTAG00000012510, ENSBTAG00000006393, ENSBTAG00000009737, ENSBTAG00000001154, ENSBTAG00000046750, ENSBTAG00000020319, ENSBTAG00000018186, ENSBTAG00000011325, ENSBTAG00000011278, ENSBTAG00000020747, ENSBTAG00000009733, ENSBTAG00000009639, ENSBTAG00000007003, ENSBTAG00000005105, ENSBTAG00000019708, ENSBTAG00000004092, ENSBTAG00000020225, ENSBTAG00000006136, ENSBTAG00000046152, ENSBTAG00000016206, ENSBTAG00000007668, ENSBTAG00000020602, ENSBTAG00000019272, ENSBTAG00000013099, ENSBTAG00000018192, ENSBTAG00000003948, ENSBTAG00000039962, ENSBTAG00000002980, ENSBTAG00000043568, ENSBTAG00000018201, ENSBTAG00000011990, ENSBTAG00000017676, ENSBTAG00000000033, ENSBTAG00000008951, ENSBTAG00000008428, ENSBTAG00000005725, ENSBTAG00000013301, ENSBTAG00000013631, ENSBTAG00000024107, ENSBTAG00000014642, ENSBTAG00000013489, ENSBTAG00000011545, ENSBTAG00000017665, ENSBTAG00000013013, ENSBTAG00000035998, ENSBTAG00000012403, ENSBTAG00000026768, ENSBTAG00000019315, ENSBTAG00000008216, ENSBTAG00000013298
**bta04668:TNF signaling pathway**	9	1.91E-02	ENSBTAG00000007191, ENSBTAG00000018200, ENSBTAG00000027513, ENSBTAG00000006143, ENSBTAG00000020676, ENSBTAG00000004211, ENSBTAG00000020936, ENSBTAG00000010007, ENSBTAG00000015727
**bta05202:Transcriptional misregulation in cancer**	12	1.97E-02	ENSBTAG00000018423, ENSBTAG00000017851, ENSBTAG00000015032, ENSBTAG00000008966, ENSBTAG00000039813, ENSBTAG00000020676, ENSBTAG00000012771, ENSBTAG00000001511, ENSBTAG00000025942, ENSBTAG00000008353, ENSBTAG00000021527, ENSBTAG00000006343
**bta00220:Arginine biosynthesis**	4	2.00E-02	ENSBTAG00000020747, ENSBTAG00000012403, ENSBTAG00000002980, ENSBTAG00000013631
**bta05144:Malaria**	6	2.39E-02	ENSBTAG00000017664, ENSBTAG00000017866, ENSBTAG00000026417, ENSBTAG00000026418, ENSBTAG00000039325, ENSBTAG00000010830
**bta00590:Arachidonic acid metabolism**	7	2.56E-02	ENSBTAG00000011990, ENSBTAG00000009737, ENSBTAG00000018281, ENSBTAG00000013298, ENSBTAG00000020319, ENSBTAG00000043553, ENSBTAG00000020225

**bta05143:African trypanosomiasis**	5	2.70E-02	ENSBTAG00000005725, ENSBTAG00000020602, ENSBTAG00000026417, ENSBTAG00000012510, ENSBTAG00000026418
**bta04670:Leukocyte transendothelial migration**	9	3.18E-02	ENSBTAG00000019627, ENSBTAG00000040432, ENSBTAG00000011473, ENSBTAG00000020676, ENSBTAG00000013451, ENSBTAG00000003176, ENSBTAG00000010007, ENSBTAG00000005077, ENSBTAG00000005055
**bta04062:Chemokine signaling pathway**	11	6.27E-02	ENSBTAG00000007191, ENSBTAG00000007193, ENSBTAG00000035587, ENSBTAG00000027513, ENSBTAG00000046320, ENSBTAG00000002923, ENSBTAG00000012510, ENSBTAG00000005077, ENSBTAG00000019428, ENSBTAG00000025257, ENSBTAG00000017839
**bta00051:Fructose and mannose metabolism**	4	7.06E-02	ENSBTAG00000006752, ENSBTAG00000011325, ENSBTAG00000013099, ENSBTAG00000009733

Supplementary file 3. Selected pathways

Immunity associated pathways and genes (H vs. control)

Gene name	Pathways	regulation status
CD274 molecule(CD274)	Cell adhesion molecules (CAMs)	D
junctional adhesion molecule 2(JAM2)	Cell adhesion molecules (CAMs)	D
intercellular adhesion molecule 1(ICAM1)	Cell adhesion molecules (CAMs)	D
programmed cell death 1(PDCD1)	Cell adhesion molecules (CAMs)	D
cytotoxic T-lymphocyte associated protein 4(CTLA4)	Cell adhesion molecules (CAMs)	D
major histocompatibility complex, class II, DQ beta(BOLA-DQB)	Cell adhesion molecules (CAMs)	D
vascular cell adhesion molecule 1-like(LOC534578)	Cell adhesion molecules (CAMs)	D
major histocompatibility complex, class II, DQ alpha 5(BOLA-DQA5)	Cell adhesion molecules (CAMs)	D
cell adhesion molecule 1(CADM1)	Cell adhesion molecules (CAMs)	U
junctional adhesion molecule 3(JAM3)	Cell adhesion molecules (CAMs)	U
versican(VCAN)	Cell adhesion molecules (CAMs)	U
nectin cell adhesion molecule 2(NECTIN2)	Cell adhesion molecules (CAMs)	U
intercellular adhesion molecule 3(ICAM3)	Cell adhesion molecules (CAMs)	U
F11 receptor(F11R)	Cell adhesion molecules (CAMs)	U
MHC class II antigen(BLA-DQB)	Cell adhesion molecules (CAMs)	U
MHC Class I JSP.1(JSP.1)	Cell adhesion molecules (CAMs)	U
selectin P ligand(SELPLG)	Cell adhesion molecules (CAMs)	U
C-X-C motif chemokine ligand 10(CXCL10)	Chemokine signaling pathway	D
C-C motif chemokine 3(LOC616364)	Chemokine signaling pathway	D
C-C motif chemokine ligand 5(CCL5)	Chemokine signaling pathway	D
signal transducer and activator of transcription 1, 91kDa(STAT1)	Chemokine signaling pathway	D
chemokine (C-C motif) ligand 8(CCL8)	Chemokine signaling pathway	D
C-C motif chemokine receptor 7(CCR7)	Chemokine signaling pathway	D
NFKB inhibitor alpha(NFKBIA)	Chemokine signaling pathway	D
C-X-C motif chemokine receptor 5(CXCR5)	Chemokine signaling pathway	D
G protein subunit beta 4(GNB4)	Chemokine signaling pathway	U
C-X3-C motif chemokine receptor 1(CX3CR1)	Chemokine signaling pathway	U
C-C motif chemokine ligand 16(CCL16)	Chemokine signaling pathway	U
HCK proto-oncogene, Src family tyrosine kinase(HCK)	Chemokine signaling pathway	U
chemokine (C-X-C motif) ligand 5(CXCL5)	Chemokine signaling pathway	U
C-C motif chemokine receptor 2(CCR2)	Chemokine signaling pathway	U
T-cell lymphoma invasion and metastasis 1(TIAM1)	Chemokine signaling pathway	U
chemokine (C-C motif) receptor 1(CCR1)	Chemokine signaling pathway	U
C-X-C motif chemokine ligand 8(CXCL8)	Chemokine signaling pathway	U
chemokine (C-X-C motif) receptor 1(CXCR1)	Chemokine signaling pathway	U
chemokine (C-X-C motif) ligand 2(CXCL2)	Chemokine signaling pathway	U
pro-platelet basic protein(PPBP)	Chemokine signaling pathway	U
arrestin beta 2(ARRB2)	Chemokine signaling pathway	U
C-X-C motif chemokine receptor 2(CXCR2)	Chemokine signaling pathway	U
thrombomodulin(THBD)	Complement and coagulation cascades	D
complement C8 gamma chain(C8G)	Complement and coagulation cascades	D
CD59 molecule(CD59)	Complement and coagulation cascades	U
complement C1q A chain(C1QA)	Complement and coagulation cascades	U
coagulation factor XIII A chain(F13A1)	Complement and coagulation cascades	U

complement C1q B chain(C1QB)	Complement and coagulation cascades	U
von Willebrand factor(VWF)	Complement and coagulation cascades	U
coagulation factor V(F5)	Complement and coagulation cascades	U
complement C5a receptor 1(C5AR1)	Complement and coagulation cascades	U
complement factor B(CFB)	Complement and coagulation cascades	U
complement factor D(CFD)	Complement and coagulation cascades	U
colony stimulating factor 1(CSF1)	Cytokine-cytokine receptor interaction	D
C-X-C motif chemokine ligand 10(CXCL10)	Cytokine-cytokine receptor interaction	D
C-C motif chemokine 3(LOC616364)	Cytokine-cytokine receptor interaction	D
TNF receptor superfamily member 25(TNFRSF25)	Cytokine-cytokine receptor interaction	D
C-C motif chemokine ligand 5(CCL5)	Cytokine-cytokine receptor interaction	D
interleukin 20 receptor subunit beta(IL20RB)	Cytokine-cytokine receptor interaction	D
chemokine (C-C motif) ligand 8(CCL8)	Cytokine-cytokine receptor interaction	D
C-C motif chemokine receptor 7(CCR7)	Cytokine-cytokine receptor interaction	D
interleukin 2 receptor subunit beta(IL2RB)	Cytokine-cytokine receptor interaction	D
atypical chemokine receptor 3(ACKR3)	Cytokine-cytokine receptor interaction	D
TNF receptor superfamily member 21(TNFRSF21)	Cytokine-cytokine receptor interaction	D
C-X-C motif chemokine receptor 5(CXCR5)	Cytokine-cytokine receptor interaction	D
tumor necrosis factor superfamily member 13(TNFSF13)	Cytokine-cytokine receptor interaction	U
interleukin 18 receptor 1(IL18R1)	Cytokine-cytokine receptor interaction	U
C-X3-C motif chemokine receptor 1(CX3CR1)	Cytokine-cytokine receptor interaction	U
TNF receptor superfamily member 1A(TNFRSF1A)	Cytokine-cytokine receptor interaction	U
interleukin 1 receptor type 1(IL1R1)	Cytokine-cytokine receptor interaction	U
transforming growth factor beta 2(TGFB2)	Cytokine-cytokine receptor interaction	U
interleukin 1 receptor type 2(IL1R2)	Cytokine-cytokine receptor interaction	U
activin A receptor type 1(ACVR1)	Cytokine-cytokine receptor interaction	U
interferon gamma receptor 1(IFNGR1)	Cytokine-cytokine receptor interaction	U
colony stimulating factor 1 receptor(CSF1R)	Cytokine-cytokine receptor interaction	U
interferon gamma receptor 2 (interferon gamma transducer 1)(IFNGR2)	Cytokine-cytokine receptor interaction	U
interleukin 1 receptor accessory protein(IL1RAP)	Cytokine-cytokine receptor interaction	U
colony stimulating factor 3 receptor(CSF3R)	Cytokine-cytokine receptor interaction	U
activin A receptor type 1B(ACVR1B)	Cytokine-cytokine receptor interaction	U
C-C motif chemokine receptor 2(CCR2)	Cytokine-cytokine receptor interaction	U
interleukin 13 receptor, alpha 1(IL13RA1)	Cytokine-cytokine receptor interaction	U
chemokine (C-C motif) receptor 1(CCR1)	Cytokine-cytokine receptor interaction	U
C-X-C motif chemokine ligand 8(CXCL8)	Cytokine-cytokine receptor interaction	U
chemokine (C-X-C motif) receptor 1(CXCR1)	Cytokine-cytokine receptor interaction	U
pro-platelet basic protein(PPBP)	Cytokine-cytokine receptor interaction	U
interleukin 18 receptor accessory protein(IL18RAP)	Cytokine-cytokine receptor interaction	U
C-X-C motif chemokine receptor 2(CXCR2)	Cytokine-cytokine receptor interaction	U
colony stimulating factor 1(CSF1)	Hematopoietic cell lineage	D
CD19 molecule(CD19)	Hematopoietic cell lineage	D
CD59 molecule(CD59)	Hematopoietic cell lineage	U
CD33 antigen-like(LOC100138951)	Hematopoietic cell lineage	U
interleukin 1 receptor type 1(IL1R1)	Hematopoietic cell lineage	U
interleukin 1 receptor type 2(IL1R2)	Hematopoietic cell lineage	U
CD1e molecule(CD1E)	Hematopoietic cell lineage	U
colony stimulating factor 1 receptor(CSF1R)	Hematopoietic cell lineage	U
colony stimulating factor 3 receptor(CSF3R)	Hematopoietic cell lineage	U
CD9 molecule(CD9)	Hematopoietic cell lineage	U
CD36 molecule(CD36)	Hematopoietic cell lineage	U

antigen-presenting glycoprotein CD1d(LOC782367)	Hematopoietic cell lineage	U
CD24 molecule(CD24)	Hematopoietic cell lineage	U
TNF alpha induced protein 3(TNFAIP3)	NF-kappa B signaling pathway	D
phospholipase C gamma 2(PLCG2)	NF-kappa B signaling pathway	D
intercellular adhesion molecule 1(ICAM1)	NF-kappa B signaling pathway	D
NFKB inhibitor alpha(NFKBIA)	NF-kappa B signaling pathway	D
p53-induced death domain protein 1(PIDD1)	NF-kappa B signaling pathway	D
B-cell linker(BLNK)	NF-kappa B signaling pathway	D
vascular cell adhesion molecule 1-like(LOC534578)	NF-kappa B signaling pathway	D
myeloid differentiation primary response 88(MYD88)	NF-kappa B signaling pathway	U
TNF receptor superfamily member 1A(TNFRSF1A)	NF-kappa B signaling pathway	U
interleukin 1 receptor type 1(IL1R1)	NF-kappa B signaling pathway	U
C-X-C motif chemokine ligand 8(CXCL8)	NF-kappa B signaling pathway	U
phospholipase C gamma 2(PLCG2)	Pathways in cancer	D
lysophosphatidic acid receptor 3(LPAR3)	Pathways in cancer	D
mutS homolog 3(MSH3)	Pathways in cancer	D
signal transducer and activator of transcription 1, 91kDa(STAT1)	Pathways in cancer	D
RAS guanyl releasing protein 3(RASGRP3)	Pathways in cancer	D
v-myc avian myelocytomatosis viral oncogene homolog(MYC)	Pathways in cancer	D
SMAD family member 3(SMAD3)	Pathways in cancer	D
baculoviral IAP repeat containing 5(BIRC5)	Pathways in cancer	D
NFKB inhibitor alpha(NFKBIA)	Pathways in cancer	D
mutL homolog 1(MLH1)	Pathways in cancer	D
fibroblast growth factor 13(FGF13)	Pathways in cancer	D
frizzled class receptor 5(FZD5)	Pathways in cancer	D
erb-b2 receptor tyrosine kinase 2(ERBB2)	Pathways in cancer	D
TNF receptor associated factor 4(TRAF4)	Pathways in cancer	D
cyclin dependent kinase 6(CDK6)	Pathways in cancer	D
peroxisome proliferator activated receptor gamma(PPARG)	Pathways in cancer	U
mutS homolog 2(MSH2)	Pathways in cancer	U
G protein subunit beta 4(GNB4)	Pathways in cancer	U
transforming growth factor beta 2(TGFB2)	Pathways in cancer	U
melanogenesis associated transcription factor(MITF)	Pathways in cancer	U
RAS guanyl releasing protein 4(RASGRP4)	Pathways in cancer	U
fibronectin 1(FN1)	Pathways in cancer	U
phosphatase and tensin homolog(PTEN)	Pathways in cancer	U
G protein subunit alpha q(GNAQ)	Pathways in cancer	U
colony stimulating factor 1 receptor(CSF1R)	Pathways in cancer	U
colony stimulating factor 3 receptor(CSF3R)	Pathways in cancer	U
peroxisome proliferator activated receptor delta(PPARD)	Pathways in cancer	U
retinoid X receptor alpha(RXRA)	Pathways in cancer	U
paired box 8(PAX8)	Pathways in cancer	U
C-X-C motif chemokine ligand 8(CXCL8)	Pathways in cancer	U
matrix metallopeptidase 9(MMP9)	Pathways in cancer	U
insulin like growth factor 1 receptor(IGF1R)	Pathways in cancer	U
carnitine palmitoyltransferase 1C(CPT1C)	PPAR signaling pathway	D
ubiquitin C(UBC)	PPAR signaling pathway	D
peroxisome proliferator activated receptor gamma(PPARG)	PPAR signaling pathway	U
oxidized low density lipoprotein receptor 1(OLR1)	PPAR signaling pathway	U
nuclear receptor subfamily 1 group H member 3(NR1H3)	PPAR signaling pathway	U

lipoprotein lipase(LPL)	PPAR signaling pathway	U
cytochrome P450, family 27, subfamily A, polypeptide 1(CYP27A1)	PPAR signaling pathway	U
peroxisome proliferator activated receptor delta(PPARD)	PPAR signaling pathway	U
retinoid X receptor alpha(RXRA)	PPAR signaling pathway	U
CD36 molecule(CD36)	PPAR signaling pathway	U
acyl-CoA synthetase long-chain family member 6(ACSL6)	PPAR signaling pathway	U
intercellular adhesion molecule 1(ICAM1)	Staphylococcus aureus infection	D
major histocompatibility complex, class II, DQ beta(BOLA-DQB)	Staphylococcus aureus infection	D
major histocompatibility complex, class II, DQ alpha 5(BOLA-DQA5)	Staphylococcus aureus infection	D
complement C1q A chain(C1QA)	Staphylococcus aureus infection	U
complement C1q B chain(C1QB)	Staphylococcus aureus infection	U
MHC class II antigen(BLA-DQB)	Staphylococcus aureus infection	U
complement C5a receptor 1(C5AR1)	Staphylococcus aureus infection	U
Fc fragment of IgA receptor(FCAR)	Staphylococcus aureus infection	U
platelet activating factor receptor(PTAFR)	Staphylococcus aureus infection	U
selectin P ligand(SELPLG)	Staphylococcus aureus infection	U
complement factor B(CFB)	Staphylococcus aureus infection	U
complement factor D(CFD)	Staphylococcus aureus infection	U
colony stimulating factor 1(CSF1)	TNF signaling pathway	D
TNF alpha induced protein 3(TNFAIP3)	TNF signaling pathway	D
C-X-C motif chemokine ligand 10(CXCL10)	TNF signaling pathway	D
C-C motif chemokine ligand 5(CCL5)	TNF signaling pathway	D
suppressor of cytokine signaling 3(SOCS3)	TNF signaling pathway	D
B-cell CLL/lymphoma 3(BCL3)	TNF signaling pathway	D
intercellular adhesion molecule 1(ICAM1)	TNF signaling pathway	D
mitogen-activated protein kinase kinase kinase 8(MAP3K8)	TNF signaling pathway	D
interferon gamma inducible protein 47(IFI47)	TNF signaling pathway	D
NFKB inhibitor alpha(NFKBIA)	TNF signaling pathway	D
vascular cell adhesion molecule 1-like(LOC534578)	TNF signaling pathway	D
interleukin 18 receptor 1(IL18R1)	TNF signaling pathway	U
TNF receptor superfamily member 1A(TNFRSF1A)	TNF signaling pathway	U
mitogen-activated protein kinase 13(MAPK13)	TNF signaling pathway	U
matrix metallopeptidase 9(MMP9)	TNF signaling pathway	U
nucleotide binding oligomerization domain containing 2(NOD2)	TNF signaling pathway	U
chemokine (C-X-C motif) ligand 2(CXCL2)	TNF signaling pathway	U

Immunity associated pathways and genes (L vs. control)

Gene names	Groups	Regulation status
colony stimulating factor 1(CSF1)	bta04060:Cytokine-cytokine receptor interaction	D
C-X-C motif chemokine ligand 10(CXCL10)	bta04060:Cytokine-cytokine receptor interaction	D
KIT proto-oncogene receptor tyrosine kinase(KIT)	bta04060:Cytokine-cytokine receptor interaction	D
tumor necrosis factor superfamily member 13(TNFSF13)	bta04060:Cytokine-cytokine receptor interaction	U
C-C motif chemokine receptor 3(CCR3)	bta04060:Cytokine-cytokine receptor interaction	U
C-X3-C motif chemokine receptor 1(CX3CR1)	bta04060:Cytokine-cytokine receptor interaction	U
transforming growth factor beta 2(TGFB2)	bta04060:Cytokine-cytokine receptor interaction	U
C-C motif chemokine ligand 5(CCL5)	bta04060:Cytokine-cytokine receptor interaction	U
C-X-C motif chemokine ligand 10(CXCL10)	bta04062:Chemokine signaling pathway	D
chemokine (C motif) ligand 1(XCL1)	bta04062:Chemokine signaling pathway	D

chemokine (C-C motif) ligand 8(CCL8)	bta04062:Chemokine signaling pathway	D
NFKB inhibitor alpha(NFKBIA)	bta04062:Chemokine signaling pathway	D
C-C motif chemokine receptor 3(CCR3)	bta04062:Chemokine signaling pathway	U
C-X3-C motif chemokine receptor 1(CX3CR1)	bta04062:Chemokine signaling pathway	U
C-C motif chemokine ligand 5(CCL5)	bta04062:Chemokine signaling pathway	U
chemokine (C-C motif) ligand 3(CCL3)	bta04062:Chemokine signaling pathway	U
chemokine (C-C motif) ligand 4(CCL4)	bta04062:Chemokine signaling pathway	U
pro-platelet basic protein(PPBP)	bta04062:Chemokine signaling pathway	U
C-X-C motif chemokine receptor 2(CXCR2)	bta04062:Chemokine signaling pathway	U
MHC class I heavy chain(BOLA)	bta04514:Cell adhesion molecules (CAMs)	D
netrin G2(NTNG2)	bta04514:Cell adhesion molecules (CAMs)	D
intercellular adhesion molecule 1(ICAM1)	bta04514:Cell adhesion molecules (CAMs)	D
L1 cell adhesion molecule(L1CAM)	bta04514:Cell adhesion molecules (CAMs)	D
contactin associated protein 1(CNTNAP1)	bta04514:Cell adhesion molecules (CAMs)	D
CD276 molecule(CD276)	bta04514:Cell adhesion molecules (CAMs)	D
vascular cell adhesion molecule 1-like(LOC534578)	bta04514:Cell adhesion molecules (CAMs)	D
major histocompatibility complex, class II, DQ alpha 5(BOLA-DQA5)	bta04514:Cell adhesion molecules (CAMs)	D
CD274 molecule(CD274)	bta04514:Cell adhesion molecules (CAMs)	D
CD4 molecule(CD4)	bta04514:Cell adhesion molecules (CAMs)	U
integrin subunit alpha L(ITGAL)	bta04514:Cell adhesion molecules (CAMs)	U
CD8b molecule(CD8B)	bta04514:Cell adhesion molecules (CAMs)	U
intercellular adhesion molecule 3(ICAM3)	bta04514:Cell adhesion molecules (CAMs)	U
cadherin 1(CDH1)	bta04514:Cell adhesion molecules (CAMs)	U
F11 receptor(F11R)	bta04514:Cell adhesion molecules (CAMs)	U
MHC class II antigen(BLA-DQB)	bta04514:Cell adhesion molecules (CAMs)	U
MHC Class I JSP.1(JSP.1)	bta04514:Cell adhesion molecules (CAMs)	U
CD8a molecule(CD8A)	bta04514:Cell adhesion molecules (CAMs)	U
sialophorin(SPN)	bta04514:Cell adhesion molecules (CAMs)	U
selectin P ligand(SELPLG)	bta04514:Cell adhesion molecules (CAMs)	U
junctional adhesion molecule 2(JAM2)	bta04514:Cell adhesion molecules (CAMs)	U
colony stimulating factor 1(CSF1)	bta04610:Complement and coagulation cascades	D
KIT proto-oncogene receptor tyrosine kinase(KIT)	bta04610:Complement and coagulation cascades	D
interleukin 9 receptor(IL9R)	bta04610:Complement and coagulation cascades	D
CD59 molecule(CD59)	bta04610:Complement and coagulation cascades	U
CD4 molecule(CD4)	bta04610:Complement and coagulation cascades	U
CD33 antigen-like(LOC100138951)	bta04610:Complement and coagulation cascades	U
CD8b molecule(CD8B)	bta04610:Complement and coagulation cascades	U
CD8a molecule(CD8A)	bta04610:Complement and coagulation cascades	U
interleukin 9 receptor(IL9R)	bta04612:Antigen processing and presentation	D
chemokine (C-C motif) ligand 8(CCL8)	bta04612:Antigen processing and presentation	D
MHC class I heavy chain(BOLA)	bta04612:Antigen processing and presentation	D
major histocompatibility complex, class II, DQ alpha 5(BOLA-DQA5)	bta04612:Antigen processing and presentation	D
C-X-C motif chemokine ligand 10(CXCL10)	bta04612:Antigen processing and presentation	D
activin A receptor type 1(ACVR1)	bta04612:Antigen processing and presentation	U
chemokine (C-C motif) ligand 3(CCL3)	bta04612:Antigen processing and presentation	U
chemokine (C-C motif) ligand 4(CCL4)	bta04612:Antigen processing and presentation	U
pro-platelet basic protein(PPBP)	bta04612:Antigen processing and presentation	U
C-X-C motif chemokine receptor 2(CXCR2)	bta04612:Antigen processing and presentation	U
CD4 molecule(CD4)	bta04612:Antigen processing and presentation	U
CD8b molecule(CD8B)	bta04612:Antigen processing and presentation	U

MHC class II antigen(BLA-DQB)	bta04612:Antigen processing and presentation	U
MHC Class I JSP.1(JSP.1)	bta04612:Antigen processing and presentation	U
CD8a molecule(CD8A)	bta04612:Antigen processing and presentation	U
Fos proto-oncogene, AP-1 transcription factor subunit(FOS)	bta04620:Toll-like receptor signaling pathway	D
mitogen-activated protein kinase kinase kinase 8(MAP3K8)	bta04620:Toll-like receptor signaling pathway	D
NFKB inhibitor alpha(NFKBIA)	bta04620:Toll-like receptor signaling pathway	D
Fas associated via death domain(FADD)	bta04620:Toll-like receptor signaling pathway	D
C-C motif chemokine ligand 5(CCL5)	bta04620:Toll-like receptor signaling pathway	U
chemokine (C-C motif) ligand 3(CCL3)	bta04620:Toll-like receptor signaling pathway	U
chemokine (C-C motif) ligand 4(CCL4)	bta04620:Toll-like receptor signaling pathway	U
complement C1q A chain(C1QA)	bta04640:Hematopoietic cell lineage	D
complement C8 gamma chain(C8G)	bta04640:Hematopoietic cell lineage	D
antigen-presenting glycoprotein CD1d(LOC782367)	bta04640:Hematopoietic cell lineage	U
complement C3d receptor 2(CR2)	bta04640:Hematopoietic cell lineage	U
CD24 molecule(CD24)	bta04640:Hematopoietic cell lineage	U
CD59 molecule(CD59)	bta04640:Hematopoietic cell lineage	U
complement C1q C chain(C1QC)	bta04640:Hematopoietic cell lineage	U
complement C1q B chain(C1QB)	bta04640:Hematopoietic cell lineage	U
serpin family G member 1(SERPING1)	bta04640:Hematopoietic cell lineage	U
complement C5a receptor 1(C5AR1)	bta04640:Hematopoietic cell lineage	U
complement C3d receptor 2(CR2)	bta04640:Hematopoietic cell lineage	U
colony stimulating factor 1(CSF1)	bta04668:TNF signaling pathway	D
TNF alpha induced protein 3(TNFAIP3)	bta04668:TNF signaling pathway	D
C-X-C motif chemokine ligand 10(CXCL10)	bta04668:TNF signaling pathway	D
Fos proto-oncogene, AP-1 transcription factor subunit(FOS)	bta04668:TNF signaling pathway	D
cAMP responsive element binding protein 3 like 1(CREB3L1)	bta04668:TNF signaling pathway	D
suppressor of cytokine signaling 3(SOCS3)	bta04668:TNF signaling pathway	D
B-cell CLL/lymphoma 3(BCL3)	bta04668:TNF signaling pathway	D
intercellular adhesion molecule 1(ICAM1)	bta04668:TNF signaling pathway	D
mitogen-activated protein kinase kinase kinase 8(MAP3K8)	bta04668:TNF signaling pathway	D
JunB proto-oncogene, AP-1 transcription factor subunit(JUNB)	bta04668:TNF signaling pathway	D
NFKB inhibitor alpha(NFKBIA)	bta04668:TNF signaling pathway	D
Fas associated via death domain(FADD)	bta04668:TNF signaling pathway	D
vascular cell adhesion molecule 1-like(LOC534578)	bta04668:TNF signaling pathway	D
C-C motif chemokine ligand 5(CCL5)	bta04668:TNF signaling pathway	U
matrix metallopeptidase 9(MMP9)	bta04668:TNF signaling pathway	U
complement C1q A chain(C1QA)	bta05150:Staphylococcus aureus infection	D
intercellular adhesion molecule 1(ICAM1)	bta05150:Staphylococcus aureus infection	D
major histocompatibility complex, class II, DQ alpha 5(BOLA-DQA5)	bta05150:Staphylococcus aureus infection	D
integrin subunit alpha L(ITGAL)	bta05150:Staphylococcus aureus infection	U
complement C1q C chain(C1QC)	bta05150:Staphylococcus aureus infection	U
complement C1q B chain(C1QB)	bta05150:Staphylococcus aureus infection	U
MHC class II antigen(BLA-DQB)	bta05150:Staphylococcus aureus infection	U
complement C5a receptor 1(C5AR1)	bta05150:Staphylococcus aureus infection	U
platelet activating factor receptor(PTAFR)	bta05150:Staphylococcus aureus infection	U
selectin P ligand(SELPLG)	bta05150:Staphylococcus aureus infection	U

Immunity associated pathways and genes (H vs. L)

Genes	Pathways	Regulation status

interleukin 5 receptor subunit alpha(IL5RA)	bta04640:Hematopoietic cell lineage	D
interleukin 1 receptor type 2(IL1R2)	bta04640:Hematopoietic cell lineage	U
interleukin 9 receptor(IL9R)	bta04640:Hematopoietic cell lineage	U
CD8b molecule(CD8B)	bta04640:Hematopoietic cell lineage	D
CD7 molecule(CD7)	bta04640:Hematopoietic cell lineage	U
CD22 molecule(CD22)	bta04640:Hematopoietic cell lineage	D
colony stimulating factor 1 receptor(CSF1R)	bta04640:Hematopoietic cell lineage	U
CD9 molecule(CD9)	bta04640:Hematopoietic cell lineage	U
CD14 molecule(CD14)	bta04640:Hematopoietic cell lineage	U
CD36 molecule(CD36)	bta04640:Hematopoietic cell lineage	U
CD8a molecule(CD8A)	bta04640:Hematopoietic cell lineage	D
complement C3d receptor 2(CR2)	bta04640:Hematopoietic cell lineage	D
CD24 molecule(CD24)	bta04640:Hematopoietic cell lineage	U
CD1a molecule-like(LOC515418)	bta04640:Hematopoietic cell lineage	D
MHC class I heavy chain(BOLA)	bta04514:Cell adhesion molecules (CAMs)	U
junctional adhesion molecule 3(JAM3)	bta04514:Cell adhesion molecules (CAMs)	U
CD8b molecule(CD8B)	bta04514:Cell adhesion molecules (CAMs)	D
CD22 molecule(CD22)	bta04514:Cell adhesion molecules (CAMs)	D
L1 cell adhesion molecule(L1CAM)	bta04514:Cell adhesion molecules (CAMs)	U
versican(VCAN)	bta04514:Cell adhesion molecules (CAMs)	U
nectin cell adhesion molecule 2(NECTIN2)	bta04514:Cell adhesion molecules (CAMs)	U
cadherin 1(CDH1)	bta04514:Cell adhesion molecules (CAMs)	D
contactin associated protein 1(CNTNAP1)	bta04514:Cell adhesion molecules (CAMs)	U
intercellular adhesion molecule 2(LOC104968494)	bta04514:Cell adhesion molecules (CAMs)	D
CD276 molecule(CD276)	bta04514:Cell adhesion molecules (CAMs)	U
CD8a molecule(CD8A)	bta04514:Cell adhesion molecules (CAMs)	D
major histocompatibility complex, class II, DQ alpha 5(BOLA-DQA5)	bta04514:Cell adhesion molecules (CAMs)	D
claudin 3(CLDN3)	bta04514:Cell adhesion molecules (CAMs)	D
interleukin 5 receptor subunit alpha(IL5RA)	bta04060:Cytokine-cytokine receptor interaction	D
C-X3-C motif chemokine receptor 1(CX3CR1)	bta04060:Cytokine-cytokine receptor interaction	D
TNF receptor superfamily member 1A(TNFRSF1A)	bta04060:Cytokine-cytokine receptor interaction	U
C-X-C motif chemokine ligand 12(CXCL12)	bta04060:Cytokine-cytokine receptor interaction	U
interleukin 1 receptor type 2(IL1R2)	bta04060:Cytokine-cytokine receptor interaction	U
C-C motif chemokine ligand 5(CCL5)	bta04060:Cytokine-cytokine receptor interaction	D
interleukin 9 receptor(IL9R)	bta04060:Cytokine-cytokine receptor interaction	U
colony stimulating factor 1 receptor(CSF1R)	bta04060:Cytokine-cytokine receptor interaction	U
interleukin 1 receptor accessory protein(IL1RAP)	bta04060:Cytokine-cytokine receptor interaction	U
activin A receptor type 1B(ACVR1B)	bta04060:Cytokine-cytokine receptor interaction	U
hepatocyte growth factor(HGF)	bta04060:Cytokine-cytokine receptor interaction	U
interleukin 15(IL15)	bta04060:Cytokine-cytokine receptor interaction	D
interleukin 13 receptor, alpha 1(IL13RA1)	bta04060:Cytokine-cytokine receptor interaction	U
chemokine (C-C motif) receptor 1(CCR1)	bta04060:Cytokine-cytokine receptor interaction	U
TNF receptor superfamily member 21(TNFRSF21)	bta04060:Cytokine-cytokine receptor interaction	D
chemokine (C-C motif) ligand 4(CCL4)	bta04060:Cytokine-cytokine receptor interaction	D
interleukin 18 receptor accessory protein(IL18RAP)	bta04060:Cytokine-cytokine receptor interaction	U
C-X-C motif chemokine receptor 5(CXCR5)	bta04060:Cytokine-cytokine receptor interaction	D
TNF receptor superfamily member 1A(TNFRSF1A)	bta04668:TNF signaling pathway	U
cAMP responsive element binding protein 3 like 1(CREB3L1)	bta04668:TNF signaling pathway	U
C-C motif chemokine ligand 5(CCL5)	bta04668:TNF signaling pathway	D

mitogen-activated protein kinase 13(MAPK13)	bta04668:TNF signaling pathway	U
interferon gamma inducible protein 47(IFI47)	bta04668:TNF signaling pathway	D
interleukin 15(IL15)	bta04668:TNF signaling pathway	D
matrix metallopeptidase 9(MMP9)	bta04668:TNF signaling pathway	U
nucleotide binding oligomerization domain containing 2(NOD2)	bta04668:TNF signaling pathway	U
chemokine (C-X-C motif) ligand 2(CXCL2)	bta04668:TNF signaling pathway	U
B-cell CLL/lymphoma 6(BCL6)	bta05202:Transcriptional misregulation in cancer	U
interleukin 1 receptor type 2(IL1R2)	bta05202:Transcriptional misregulation in cancer	U
cyclin dependent kinase inhibitor 1A(CDKN1A)	bta05202:Transcriptional misregulation in cancer	U
tetraspanin 7(TSPAN7)	bta05202:Transcriptional misregulation in cancer	U
colony stimulating factor 1 receptor(CSF1R)	bta05202:Transcriptional misregulation in cancer	U
CD14 molecule(CD14)	bta05202:Transcriptional misregulation in cancer	U
retinoid X receptor alpha(RXRA)	bta05202:Transcriptional misregulation in cancer	U
DEAD-box helicase 5(DDX5)	bta05202:Transcriptional misregulation in cancer	D
matrix metallopeptidase 9(MMP9)	bta05202:Transcriptional misregulation in cancer	U
insulin like growth factor 1 receptor(IGF1R)	bta05202:Transcriptional misregulation in cancer	U
hydroxyprostaglandin dehydrogenase 15-(NAD)(HPGD)	bta05202:Transcriptional misregulation in cancer	D
granzyme B (granzyme 2, cytotoxic T-lymphocyte-associated serine esterase 1)(GZMB)	bta05202:Transcriptional misregulation in cancer	D

D: Down-regulated
U: Up-regulated

Supplementary file 4. Common genes enriched in pathways

Groups	Total	Genes ID
high vs control high vs low low vs control	5	ENSBTAG00000020676
		ENSBTAG00000007191
		ENSBTAG00000038128
		ENSBTAG00000002923
		ENSBTAG00000039046
high vs control low vs control	30	ENSBTAG00000000095
		ENSBTAG00000038042
		ENSBTAG00000015742
		ENSBTAG00000009493
		ENSBTAG00000037539
		ENSBTAG00000010520
		ENSBTAG00000010303
		ENSBTAG00000002302
		ENSBTAG00000007153
		ENSBTAG00000005359
		ENSBTAG00000032425
		ENSBTAG00000000130
		ENSBTAG00000017846
		ENSBTAG00000001725
		ENSBTAG00000027051
		ENSBTAG00000004608
		ENSBTAG00000024960
		ENSBTAG00000014113
		ENSBTAG00000019588
		ENSBTAG00000011600
		ENSBTAG00000016683
		ENSBTAG00000031851
		ENSBTAG00000000436
		ENSBTAG00000020872
		ENSBTAG00000011196
		ENSBTAG00000008441
		ENSBTAG00000000283
		ENSBTAG00000000603
		ENSBTAG00000020116
		ENSBTAG00000011909
high vs control high vs low	21	ENSBTAG00000033748
		ENSBTAG00000015318
		ENSBTAG00000006343

		ENSBTAG00000003176
		ENSBTAG00000014764
		ENSBTAG00000004211
		ENSBTAG00000013555
		ENSBTAG00000020936
		ENSBTAG00000046320
		ENSBTAG00000019338
		ENSBTAG00000012771
		ENSBTAG00000010007
		ENSBTAG00000014906
		ENSBTAG00000017866
		ENSBTAG00000021527
		ENSBTAG00000013205
		ENSBTAG00000027513
		ENSBTAG00000017851
		ENSBTAG00000019428
		ENSBTAG00000015727
		ENSBTAG00000020054
high vs low low vs control	11	ENSBTAG00000007558
		ENSBTAG00000038496
		ENSBTAG00000008956
		ENSBTAG00000019097
		ENSBTAG00000021141
		ENSBTAG00000006143
		ENSBTAG00000025257
		ENSBTAG00000015991
		ENSBTAG00000002069
		ENSBTAG00000013462
		ENSBTAG00000019734
high vs control	66	ENSBTAG00000019708
		ENSBTAG00000013334
		ENSBTAG00000044023
		ENSBTAG00000046158
		ENSBTAG00000012599
		ENSBTAG00000013170
		ENSBTAG00000001333
		ENSBTAG00000021077
		ENSBTAG00000048122
		ENSBTAG00000010681
		ENSBTAG00000001034

ENSBTAG00000002758
ENSBTAG00000021798
ENSBTAG00000006679
ENSBTAG00000007268
ENSBTAG00000019354
ENSBTAG00000017542
ENSBTAG00000008409
ENSBTAG00000021358
ENSBTAG00000004547
ENSBTAG00000021647
ENSBTAG00000013573
ENSBTAG00000013586
ENSBTAG00000016758
ENSBTAG00000035587
ENSBTAG00000017839
ENSBTAG00000019716
ENSBTAG00000009789
ENSBTAG00000002103
ENSBTAG00000016345
ENSBTAG00000012855
ENSBTAG00000007932
ENSBTAG00000012544
ENSBTAG00000000977
ENSBTAG00000005273
ENSBTAG00000007193
ENSBTAG00000011543
ENSBTAG00000009421
ENSBTAG00000008300
ENSBTAG00000017722
ENSBTAG00000008006
ENSBTAG00000006998
ENSBTAG00000018424
ENSBTAG00000007684
ENSBTAG00000015133
ENSBTAG00000019634
ENSBTAG00000006519
ENSBTAG00000002767
ENSBTAG00000007867
ENSBTAG00000009812
ENSBTAG00000003289

		ENSBTAG00000003791
		ENSBTAG00000038990
		ENSBTAG00000009498
		ENSBTAG00000012265
		ENSBTAG00000012899
		ENSBTAG00000026753
		ENSBTAG00000032436
		ENSBTAG00000032122
		ENSBTAG00000013489
		ENSBTAG00000020957
		ENSBTAG00000012205
		ENSBTAG00000000563
		ENSBTAG00000020194
		ENSBTAG00000008299
		ENSBTAG00000002742
low vs control	13	ENSBTAG00000009938
		ENSBTAG00000003255
		ENSBTAG00000018274
		ENSBTAG00000026326
		ENSBTAG00000016267
		ENSBTAG00000012046
		ENSBTAG00000007103
		ENSBTAG00000011193
		ENSBTAG00000007235
		ENSBTAG00000025250
		ENSBTAG00000001338
		ENSBTAG00000002699
		ENSBTAG00000004322
high vs low	16	ENSBTAG00000008966
		ENSBTAG00000017664
		ENSBTAG00000005077
		ENSBTAG00000015032
		ENSBTAG00000040432
		ENSBTAG00000039366
		ENSBTAG00000019432
		ENSBTAG00000008353
		ENSBTAG00000000273
		ENSBTAG00000039813
		ENSBTAG00000001511
		ENSBTAG00000011887

	ENSBTAG00000018423
	ENSBTAG00000018200
	ENSBTAG00000025942
	ENSBTAG00000011359

Supplementary file 5. Biological function processes

High folic acid treated group regulated biological processes

Term	Count	*P -value*
GO:0006955~immune response	36	1.95E-08
GO:0006954~inflammatory response	33	6.42E-07
GO:0030593~neutrophil chemotaxis	14	1.51E-06
GO:0070098~chemokine-mediated signaling pathway	13	4.49E-06
GO:0002523~leukocyte migration involved in inflammatory response	6	1.17E-05
GO:0031623~receptor internalization	9	2.14E-04
GO:0006935~chemotaxis	11	3.88E-04
GO:0070374~positive regulation of ERK1 and ERK2 cascade	19	4.42E-04
GO:0006919~activation of cysteine-type endopeptidase activity involved in apoptotic process	10	8.81E-04
GO:0048661~positive regulation of smooth muscle cell proliferation	7	9.02E-04
GO:0090026~positive regulation of monocyte chemotaxis	6	1.14E-03
GO:0098869~cellular oxidant detoxification	8	1.56E-03
GO:0042742~defense response to bacterium	13	2.14E-03
GO:0007596~blood coagulation	10	2.22E-03
GO:0032496~response to lipopolysaccharide	13	2.64E-03
GO:0048662~negative regulation of smooth muscle cell proliferation	6	2.81E-03
GO:0006915~apoptotic process	23	3.25E-03
GO:0050900~leukocyte migration	6	3.63E-03
GO:0043066~negative regulation of apoptotic process	26	3.95E-03
GO:0042127~regulation of cell proliferation	18	4.21E-03
GO:0007155~cell adhesion	20	4.92E-03
GO:0019370~leukotriene biosynthetic process	4	8.47E-03
GO:0050729~positive regulation of inflammatory response	8	9.25E-03
GO:0002292~T cell differentiation involved in immune response	3	9.48E-03
GO:0038094~Fc-gamma receptor signaling pathway	3	9.48E-03
GO:0001525~angiogenesis	16	9.87E-03
GO:0009615~response to virus	8	1.19E-02
GO:1902042~negative regulation of extrinsic apoptotic signaling pathway via death domain receptors	5	1.40E-02
GO:0043123~positive regulation of I-kappaB kinase/NF-kappaB signaling	15	1.41E-02
GO:0042060~wound healing	7	1.56E-02
GO:0030889~negative regulation of B cell proliferation	4	1.67E-02
GO:0032495~response to muramyl dipeptide	4	1.67E-02
GO:0097067~cellular response to thyroid hormone stimulus	4	1.67E-02
GO:0060326~cell chemotaxis	8	1.69E-02
GO:0050715~positive regulation of cytokine secretion	5	1.72E-02
GO:0090307~mitotic spindle assembly	6	2.01E-02
GO:0045087~innate immune response	22	2.09E-02
GO:0006979~response to oxidative stress	9	2.26E-02
GO:0019882~antigen processing and presentation	6	2.31E-02
GO:0045766~positive regulation of angiogenesis	11	2.33E-02
GO:0021762~substantia nigra development	6	2.65E-02
GO:0007010~cytoskeleton organization	10	2.72E-02
GO:0007076~mitotic chromosome condensation	4	2.80E-02

GO:0030193~regulation of blood coagulation	4	2.80E-02
GO:0002690~positive regulation of leukocyte chemotaxis	4	2.80E-02
GO:0030574~collagen catabolic process	4	2.80E-02
GO:0006911~phagocytosis, engulfment	4	2.80E-02
GO:0042119~neutrophil activation	3	2.93E-02
GO:0001913~T cell mediated cytotoxicity	3	2.93E-02
GO:0032494~response to peptidoglycan	3	2.93E-02
GO:1900746~regulation of vascular endothelial growth factor signaling pathway	3	2.93E-02
GO:0090501~RNA phosphodiester bond hydrolysis	3	2.93E-02
GO:0070234~positive regulation of T cell apoptotic process	3	2.93E-02
GO:0048247~lymphocyte chemotaxis	5	3.44E-02
GO:0046579~positive regulation of Ras protein signal transduction	4	3.49E-02
GO:0006402~mRNA catabolic process	4	3.49E-02
GO:0007050~cell cycle arrest	8	4.00E-02
GO:0050862~positive regulation of T cell receptor signaling pathway	3	4.22E-02
GO:0046007~negative regulation of activated T cell proliferation	3	4.22E-02
GO:0070233~negative regulation of T cell apoptotic process	3	4.22E-02
GO:0010886~positive regulation of cholesterol storage	3	4.22E-02
GO:0045190~isotype switching	4	4.26E-02
GO:0071356~cellular response to tumor necrosis factor	8	4.71E-02
GO:0055085~transmembrane transport	12	5.07E-02
GO:0030308~negative regulation of cell growth	10	5.09E-02
GO:0001974~blood vessel remodeling	5	5.20E-02
GO:0071346~cellular response to interferon-gamma	6	5.30E-02
GO:0016310~phosphorylation	6	5.30E-02
GO:0007288~sperm axoneme assembly	3	5.69E-02
GO:0035357~peroxisome proliferator activated receptor signaling pathway	3	5.69E-02
GO:0050702~interleukin-1 beta secretion	3	5.69E-02
GO:0000122~negative regulation of transcription from RNA polymerase II promoter	33	6.18E-02
GO:0006974~cellular response to DNA damage stimulus	12	6.25E-02
GO:0071456~cellular response to hypoxia	7	6.51E-02
GO:0050821~protein stabilization	11	6.74E-02
GO:0042832~defense response to protozoan	4	7.01E-02
GO:0050727~regulation of inflammatory response	6	7.05E-02
GO:0050892~intestinal absorption	3	7.31E-02
GO:0071404~cellular response to low-density lipoprotein particle stimulus	3	7.31E-02
GO:0010744~positive regulation of macrophage derived foam cell differentiation	3	7.31E-02
GO:1900017~positive regulation of cytokine production involved in inflammatory response	3	7.31E-02
GO:0045806~negative regulation of endocytosis	3	7.31E-02
GO:0031663~lipopolysaccharide-mediated signaling pathway	5	7.37E-02
GO:0032755~positive regulation of interleukin-6 production	6	7.69E-02
GO:0043401~steroid hormone mediated signaling pathway	4	8.07E-02
GO:0001816~cytokine production	4	8.07E-02
GO:0007611~learning or memory	4	8.07E-02
GO:0032733~positive regulation of interleukin-10 production	4	8.07E-02
GO:0002548~monocyte chemotaxis	5	8.18E-02
GO:0006816~calcium ion transport	6	8.37E-02

GO:0010880~regulation of release of sequestered calcium ion into cytosol by sarcoplasmic reticulum	3	9.05E-02
GO:0032703~negative regulation of interleukin-2 production	3	9.05E-02
GO:0008015~blood circulation	3	9.05E-02
GO:0006750~glutathione biosynthetic process	3	9.05E-02
GO:0002376~immune system process	3	9.05E-02
GO:0043303~mast cell degranulation	3	9.05E-02
GO:0034599~cellular response to oxidative stress	6	9.07E-02
GO:0090023~positive regulation of neutrophil chemotaxis	4	9.19E-02
GO:0032715~negative regulation of interleukin-6 production	4	9.19E-02
GO:0032508~DNA duplex unwinding	4	9.19E-02
GO:0050728~negative regulation of inflammatory response	7	9.38E-02
GO:0007507~heart development	11	9.53E-02
GO:0010862~positive regulation of pathway-restricted SMAD protein phosphorylation	6	9.81E-02
GO:0007080~mitotic metaphase plate congression	5	9.92E-02

Low folic acid treated group regulated biological processes

Term	Count	P-value
GO:0019370~leukotriene biosynthetic process	6	8.21E-07
GO:0050850~positive regulation of calcium-mediated signaling	6	5.33E-05
GO:0050900~leukocyte migration	6	1.33E-04
GO:0070098~chemokine-mediated signaling pathway	8	1.85E-04
GO:0030593~neutrophil chemotaxis	8	2.82E-04
GO:0006955~immune response	17	3.19E-04
GO:0006954~inflammatory response	17	3.19E-04
GO:0043001~Golgi to plasma membrane protein transport	5	1.96E-03
GO:0048247~lymphocyte chemotaxis	5	2.83E-03
GO:0071346~cellular response to interferon-gamma	6	2.92E-03
GO:0070374~positive regulation of ERK1 and ERK2 cascade	11	3.25E-03
GO:0007166~cell surface receptor signaling pathway	12	3.50E-03
GO:2000503~positive regulation of natural killer cell chemotaxis	3	4.40E-03
GO:0071356~cellular response to tumor necrosis factor	7	5.16E-03
GO:0050729~positive regulation of inflammatory response	6	5.76E-03
GO:0002548~monocyte chemotaxis	5	7.89E-03
GO:0071347~cellular response to interleukin-1	6	9.29E-03
GO:0006935~chemotaxis	6	1.10E-02
GO:0006906~vesicle fusion	6	1.30E-02
GO:0001775~cell activation	3	1.46E-02
GO:0006909~phagocytosis	5	1.53E-02
GO:0010628~positive regulation of gene expression	10	1.71E-02
GO:2000352~negative regulation of endothelial cell apoptotic process	4	2.18E-02
GO:0007155~cell adhesion	11	2.20E-02
GO:0006508~proteolysis	11	2.36E-02
GO:0045087~innate immune response	13	2.41E-02
GO:0001817~regulation of cytokine production	3	2.41E-02
GO:0010759~positive regulation of macrophage chemotaxis	3	2.96E-02

GO:0045766~positive regulation of angiogenesis	7	3.01E-02
GO:0009615~response to virus	5	3.27E-02
GO:0045165~cell fate commitment	5	3.51E-02
GO:1903352~L-ornithine transmembrane transport	3	3.55E-02
GO:1900745~positive regulation of p38MAPK cascade	3	3.55E-02
GO:0006887~exocytosis	5	3.77E-02
GO:0031663~lipopolysaccharide-mediated signaling pathway	4	4.12E-02
GO:0048565~digestive tract development	4	4.12E-02
GO:0002690~positive regulation of leukocyte chemotaxis	3	4.19E-02
GO:0043922~negative regulation by host of viral transcription	3	4.86E-02
GO:0060707~trophoblast giant cell differentiation	3	4.86E-02
GO:0071277~cellular response to calcium ion	4	4.90E-02
GO:0048791~calcium ion-regulated exocytosis of neurotransmitter	4	5.32E-02
GO:0060374~mast cell differentiation	2	5.45E-02
GO:0061290~canonical Wnt signaling pathway involved in metanephric kidney development	2	5.45E-02
GO:0032507~maintenance of protein location in cell	2	5.45E-02
GO:0002540~leukotriene production involved in inflammatory response	2	5.45E-02
GO:0023014~signal transduction by protein phosphorylation	3	5.57E-02
GO:0051928~positive regulation of calcium ion transport	3	5.57E-02
GO:0071407~cellular response to organic cyclic compound	4	6.20E-02
GO:0050832~defense response to fungus	3	6.31E-02
GO:0050728~negative regulation of inflammatory response	5	6.51E-02
GO:0009314~response to radiation	3	7.08E-02
GO:0045773~positive regulation of axon extension	3	7.08E-02
GO:0009636~response to toxic substance	4	7.15E-02
GO:0007204~positive regulation of cytosolic calcium ion concentration	5	7.23E-02
GO:0032496~response to lipopolysaccharide	6	7.96E-02
GO:0043066~negative regulation of apoptotic process	12	8.00E-02
GO:2000269~regulation of fibroblast apoptotic process	2	8.06E-02
GO:0034140~negative regulation of toll-like receptor 3 signaling pathway	2	8.06E-02
GO:0014070~response to organic cyclic compound	2	8.06E-02
GO:1904468~negative regulation of tumor necrosis factor secretion	2	8.06E-02
GO:0061179~negative regulation of insulin secretion involved in cellular response to glucose stimulus	2	8.06E-02
GO:0003006~developmental process involved in reproduction	2	8.06E-02
GO:0000453~enzyme-directed rRNA 2'-O-methylation	2	8.06E-02
GO:0045415~negative regulation of interleukin-8 biosynthetic process	2	8.06E-02
GO:0010871~negative regulation of receptor biosynthetic process	2	8.06E-02
GO:1900122~positive regulation of receptor binding	2	8.06E-02
GO:0001878~response to yeast	2	8.06E-02
GO:0042518~negative regulation of tyrosine phosphorylation of Stat3 protein	2	8.06E-02
GO:0002291~T cell activation via T cell receptor contact with antigen bound to MHC molecule on antigen presenting cell	2	8.06E-02
GO:0048538~thymus development	4	8.16E-02

GO:0048662~negative regulation of smooth muscle cell proliferation	3	8.71E-02
GO:0031295~T cell costimulation	3	8.71E-02
GO:0045600~positive regulation of fat cell differentiation	4	9.23E-02
GO:0032689~negative regulation of interferon-gamma production	3	9.57E-02
GO:0034198~cellular response to amino acid starvation	3	9.57E-02
GO:0046580~negative regulation of Ras protein signal transduction	3	9.57E-02
GO:0006869~lipid transport	4	9.78E-02

High vs. low folic acid treated group regulated biological processes

Term	Count	P-value
GO:0006954~inflammatory response	21	3.36E-05
GO:0006955~immune response	19	3.09E-04
GO:0098869~cellular oxidant detoxification	7	4.76E-04
GO:0002523~leukocyte migration involved in inflammatory response	4	1.16E-03
GO:0090026~positive regulation of monocyte chemotaxis	5	1.23E-03
GO:0019370~leukotriene biosynthetic process	4	1.81E-03
GO:0030308~negative regulation of cell growth	10	1.87E-03
GO:0050729~positive regulation of inflammatory response	7	2.49E-03
GO:0070098~chemokine-mediated signaling pathway	7	3.17E-03
GO:0016310~phosphorylation	6	6.42E-03
GO:0042771~intrinsic apoptotic signaling pathway in response to DNA damage by p53 class mediator	5	6.48E-03
GO:0043922~negative regulation by host of viral transcription	4	8.16E-03
GO:0045087~innate immune response	16	9.07E-03
GO:0042127~regulation of cell proliferation	12	9.60E-03
GO:0070374~positive regulation of ERK1 and ERK2 cascade	11	1.15E-02
GO:0051289~protein homotetramerization	6	1.36E-02
GO:0050850~positive regulation of calcium-mediated signaling	4	1.48E-02
GO:1900165~negative regulation of interleukin-6 secretion	3	1.52E-02
GO:0031623~receptor internalization	5	1.67E-02
GO:0030318~melanocyte differentiation	4	1.76E-02
GO:0042742~defense response to bacterium	8	1.81E-02
GO:0055114~oxidation-reduction process	19	1.90E-02
GO:0030593~neutrophil chemotaxis	6	1.94E-02
GO:0051607~defense response to virus	10	2.37E-02
GO:0007596~blood coagulation	6	2.67E-02
GO:0051260~protein homooligomerization	10	2.96E-02
GO:0008285~negative regulation of cell proliferation	14	3.00E-02
GO:0008630~intrinsic apoptotic signaling pathway in response to DNA damage	5	3.08E-02
GO:0048661~positive regulation of smooth muscle cell proliferation	4	3.12E-02
GO:0032729~positive regulation of interferon-gamma production	5	3.36E-02
GO:0038083~peptidyl-tyrosine autophosphorylation	5	3.98E-02
GO:0072657~protein localization to membrane	3	4.98E-02
GO:0051017~actin filament bundle assembly	4	5.40E-02
GO:0043085~positive regulation of catalytic activity	4	5.40E-02
GO:0009615~response to virus	5	5.78E-02
GO:0007169~transmembrane receptor protein tyrosine kinase signaling pathway	6	6.14E-02
GO:0030335~positive regulation of cell migration	9	6.16E-02

GO:0070488~neutrophil aggregation	2	6.54E-02
GO:0002793~positive regulation of peptide secretion	2	6.54E-02
GO:0015939~pantothenate metabolic process	2	6.54E-02
GO:0002540~leukotriene production involved in inflammatory response	2	6.54E-02
GO:2000660~negative regulation of interleukin-1-mediated signaling pathway	2	6.54E-02
GO:0007264~small GTPase mediated signal transduction	12	6.90E-02
GO:0006914~autophagy	5	7.03E-02
GO:0071277~cellular response to calcium ion	4	7.66E-02
GO:0051928~positive regulation of calcium ion transport	3	7.72E-02
GO:0045190~isotype switching	3	7.72E-02
GO:0048791~calcium ion-regulated exocytosis of neurotransmitter	4	8.28E-02
GO:0032720~negative regulation of tumor necrosis factor production	4	8.28E-02
GO:0006461~protein complex assembly	4	8.28E-02
GO:0046718~viral entry into host cell	3	8.72E-02
GO:0006906~vesicle fusion	5	8.91E-02
GO:0042552~myelination	4	8.92E-02
GO:0001666~response to hypoxia	6	9.56E-02
GO:0032386~regulation of intracellular transport	2	9.65E-02
GO:0038094~Fc-gamma receptor signaling pathway	2	9.65E-02
GO:0019064~fusion of virus membrane with host plasma membrane	2	9.65E-02
GO:0006768~biotin metabolic process	2	9.65E-02
GO:0032596~protein transport into membrane raft	2	9.65E-02
GO:0002292~T cell differentiation involved in immune response	2	9.65E-02
GO:0051491~positive regulation of filopodium assembly	3	9.75E-02

Supplementary file 6. Distribution of genes in biological function processes

GO TERM	Function (High vs. control)	Total	Up	Down	*P-value*
GO:0006955	immune response	36	15	21	1.90E-08
GO:0006954	inflammatory response	33	24	9	6.40E-07
GO:0030593	neutrophil chemotaxis	14	9	5	1.50E-06
GO:0070098	chemokine-mediated signaling pathway	13	7	6	4.50E-06
GO:0002523	leukocyte migration involved in inflammatory response	6	6		1.20E-05
GO:0031623	receptor internalization	9	4	5	2.10E-04
GO:0006935	chemotaxis	11		11	3.90E-04
GO:0070374	positive regulation of ERK1 and ERK2 cascade	19	11	8	4.40E-04
GO:0006919	activation of cysteine-type endopeptidase activity involved in apoptotic process	10	8	2	8.80E-04
GO:0048661	positive regulation of smooth muscle cell proliferation	7	6	1	9.00E-04
GO:0090026	positive regulation of monocyte chemotaxis	6	5	1	1.10E-03
GO:0098869	cellular oxidant detoxification	8	2	6	1.60E-03
GO:0042742	defense response to bacterium	13	13		2.10E-03
GO:0007596	blood coagulation	10	5	5	2.20E-03
GO:0048662	negative regulation of smooth muscle cell proliferation	6	6		2.80E-03
GO:0006915	apoptotic process	23	20	3	3.30E-03
GO:0050900	leukocyte migration	6	3	3	3.60E-03
GO:0043066	negative regulation of apoptotic process	26	12	14	4.00E-03
GO:0042127	regulation of cell proliferation	18	12	6	4.20E-03
GO:0007155	cell adhesion	20	13	7	4.90E-03
GO:0019370	leukotriene biosynthetic process	4	3	1	8.50E-03
GO:0050729	positive regulation of inflammatory response	8	5	3	9.30E-03
GO:0038094	Fc-gamma receptor signaling pathway	3	3		9.50E-03
GO:0002292	T cell differentiation involved in immune response	3	2	1	9.50E-03
GO:0009615	response to virus	8	4	4	1.20E-02
GO:1902042	negative regulation of extrinsic apoptotic signaling pathway via death domain receptors	5	2	3	1.40E-02
GO:0043123	positive regulation of I-kappaB kinase/NF-kappaB signaling	15	14	1	1.40E-02
GO:0042060	wound healing	7	5	2	1.60E-02
GO:0030889	negative regulation of B cell proliferation	4	3	1	1.70E-02
GO:0097067	cellular response to thyroid hormone stimulus	4	2	2	1.70E-02
GO:0032495	response to muramyl dipeptide	4	4		1.70E-02
GO:0060326	cell chemotaxis	8	5	3	1.70E-02
GO:0050715	positive regulation of cytokine secretion	5	5		1.70E-02
GO:0045087	innate immune response	22	13	9	2.10E-02
GO:0006979	response to oxidative stress	9	7	2	2.30E-02
GO:0019882	antigen processing and presentation	6	5	1	2.30E-02
GO:0045766~	positive regulation of angiogenesis	11	8	3	2.30E-02
GO:0030193	regulation of blood coagulation	4	4		2.80E-02
GO:0002690	positive regulation of leukocyte chemotaxis	4	4		2.80E-02

GO:0006911	phagocytosis, engulfment	4		4	2.80E-02
GO:0030574	collagen catabolic process	4	3	1	2.80E-02
GO:0001913	T cell mediated cytotoxicity	3	3		2.90E-02
GO:0042119	neutrophil activation	3	1	2	2.90E-02
GO:1900746	regulation of vascular endothelial growth factor signaling pathway	3	1	2	2.90E-02
GO:0070234	positive regulation of T cell apoptotic process	3	1	2	2.90E-02
GO:0048247	lymphocyte chemotaxis	5		5	3.40E-02
GO:0046007	negative regulation of activated T cell proliferation	3	3		4.20E-02
GO:0050862	positive regulation of T cell receptor signaling pathway	3	1	2	4.20E-02
GO:0070233	negative regulation of T cell apoptotic process	3	2	1	4.20E-02
GO:0071356	cellular response to tumor necrosis factor	8	4	4	4.70E-02

GO TERM	Biological function processes (low vs. control)	up-regu	down-re	total	p value
GO:0019370	leukotriene biosynthetic process	3	3	6	8.20E-07
GO:0050900	leukocyte migration	4	2	6	1.30E-04
GO:0070098	chemokine-mediated signaling pathway	6	2	8	1.80E-04
GO:0030593	neutrophil chemotaxis	7	1	8	2.80E-04
GO:0006954	immune response	9	8	17	3.20E-04
GO:0006955	inflammatory response	10	7	17	3.20E-04
GO:0048247	lymphocyte chemotaxis	4	1	5	2.80E-03
GO:0071346	cellular response to interferon-gamma	4	2	6	2.90E-03
GO:0070374	positive regulation of ERK1 and ERK2 cascade	8	3	11	3.20E-03
GO:0007166	cell surface receptor signaling pathway	10	2	12	3.50E-03
GO:2000503	positive regulation of natural killer cell chemotaxis	3		3	4.40E-03
GO:0071356	cellular response to tumor necrosis factor	4	3	7	5.20E-03
GO:0050729	positive regulation of inflammatory response	4	2	6	5.80E-03
GO:0002548	monocyte chemotaxis	4	1	5	7.90E-03
GO:0071347	cellular response to interleukin-1	4	2	6	9.30E-03
GO:0006935	chemotaxis	4	2	6	1.10E-02
GO:0001775	cell activation	3		3	1.50E-02
GO:0006909	phagocytosis	5		5	1.50E-02
GO:0010628	positive regulation of gene expression	6	4	10	1.70E-02
GO:2000352	negative regulation of endothelial cell apoptotic process	1	3	4	2.20E-02
GO:0007155	cell adhesion	7	4	11	2.20E-02
GO:0045087	innate immune response	7	6	13	2.40E-02
GO:0001817	regulation of cytokine production	2	1	3	2.40E-02
GO:0010759	positive regulation of macrophage chemotaxis	2	1	3	3.00E-02
GO:0009615	response to virus	2	3	5	3.30E-02
GO:0045165	cell fate commitment	1	4	5	3.50E-02
GO:1900745	positive regulation of p38MAPK cascade		3	3	3.60E-02
GO:0002690	positive regulation of leukocyte chemotaxis	2	1	3	4.20E-02

GO:0060707	trophoblast giant cell differentiation	3		3	4.90E-02
GO:0043922	negative regulation by host of viral transcription		3	3	4.90E-02

Biological function	Up	Down	Genes hit	*P-value*
inflammatory response	16	5	21	3.40E-05
immune response	10	9	19	3.10E-04
cellular oxidant detoxification	6	1	7	4.80E-04
leukocyte migration involved in inflammatory response	4		4	1.20E-03
positive regulation of monocyte chemotaxis	4	1	5	1.20E-03
leukotriene biosynthetic process	3	1	4	1.80E-03
negative regulation of cell growth	6	4	10	1.90E-03
positive regulation of inflammatory response	4	3	7	2.50E-03
chemokine-mediated signaling pathway	3	4	7	3.20E-03
phosphorylation	3	3	6	6.40E-03
intrinsic apoptotic signaling pathway in response to DNA damage by p53 class mediator	3	2	5	6.50E-03
negative regulation by host of viral transcription	1	3	4	8.20E-03
innate immune response	12	4	16	9.10E-03
regulation of cell proliferation	9	3	12	9.60E-03
positive regulation of ERK1 and ERK2 cascade	8	3	11	1.20E-02
positive regulation of calcium-mediated signaling	2	2	4	1.50E-02
negative regulation of interleukin-6 secretion	2	1	3	1.50E-02
receptor internalization	5		5	1.70E-02
melanocyte differentiation	2	2	4	1.80E-02
defense response to bacterium	8		8	1.80E-02
neutrophil chemotaxis	3	3	6	1.90E-02
defense response to virus	5	5	10	2.40E-02
blood coagulation	5	1	6	2.70E-02
negative regulation of cell proliferation	10	4	14	3.00E-02
intrinsic apoptotic signaling pathway in response to DNA damage	1	4	5	3.10E-02
positive regulation of interferon-gamma production	2	3	5	3.40E-02

Supplementary file 7. Differentially expressed genes

DEGs regulated by high folic acid treatment

Gene ID	Gene name	Sample_1	Sample_2	Value_1	Value_2	Expression	Log2(fold_change)	P-value
ENSBTAG00000020054	TNFRSF21	A	C	30.3581	53.8832	downregulated	0.827753	0.00195
ENSBTAG00000046320	CXCR5	A	C	14.9812	26.8321	downregulated	0.840802	0.0099
ENSBTAG00000010007	MAPK13	A	C	6.212	2.30188	upregulated	-1.43225	0.0007
ENSBTAG00000048122	CFD	A	C	1950.06	924.571	upregulated	-1.07666	0.00395
ENSBTAG00000033748	IL18RAP	A	C	4.90047	2.27766	upregulated	-1.10537	0.00675
ENSBTAG00000027513	CXCL2	A	C	3.10723	0.937682	upregulated	-1.72846	0.008
ENSBTAG00000020936	NOD2	A	C	4.92626	2.83043	upregulated	-0.799474	0.0089
ENSBTAG00000026753	CXCR1	A	C	71.3766	42.0987	upregulated	-0.761676	0.01165
ENSBTAG00000046158	CFB	A	C	0.829592	0.341589	upregulated	-1.28014	0.03625
ENSBTAG00000000563	MyD88	A	C	118.068	80.2043	upregulated	-0.557863	0.04175

DEGs regulated by low folic acid treatment

Gene ID	Gene name	Sample_1	Sample_2	Value_1	Value_2	Expression	Log2(fold_change)	P-value
ENSBTAG00000038128	BOLA-DQA5	B	C	22.0171	335.876	downregulated	3.93123	5.00E-05
ENSBTAG00000008441	SOCS3	B	C	26.6102	74.5781	downregulated	1.48678	5.00E-05
ENSBTAG00000001725	CXCL10	B	C	13.7634	34.0148	downregulated	1.30532	0.0002
ENSBTAG00000014113	CCL8	B	C	0.652584	5.19669	downregulated	2.99336	0.00135
ENSBTAG00000016683	NFKBIA	B	C	92.7742	166.468	downregulated	0.843452	0.00465
ENSBTAG00000004322	FOS	B	C	19.5567	32.2976	downregulated	0.723765	0.0061
ENSBTAG00000018274	FADD	B	C	68.9504	96.1903	downregulated	0.480333	0.0301
ENSBTAG00000002699	KIT	B	C	1.16943	1.85904	downregulated	0.66875	0.03685
ENSBTAG00000000283	CSF1	B	C	0.824507	1.6442	downregulated	0.995784	0.04545
ENSBTAG00000020116	JSP.1	B	C	191.113	18.5687	upregulated	-3.36348	5.00E-05
ENSBTAG00000019588	BLA-DQB	B	C	1263.13	541.071	upregulated	-1.22312	0.0003
ENSBTAG00000021141	CD8A	B	C	45.9677	26.2183	upregulated	-0.810045	0.00055
ENSBTAG00000003255	CD4	B	C	166.204	99.2697	upregulated	-0.743529	0.00115
ENSBTAG00000032425	PPBP	B	C	176.966	101.16	upregulated	-0.806837	0.00185
ENSBTAG00000025257	CCL4	B	C	22.019	10.8534	upregulated	-1.02061	0.00545
ENSBTAG00000038042	CXCR2	B	C	77.0515	49.6174	upregulated	-0.634978	0.01015
ENSBTAG00000025250	CCL3	B	C	8.22274	4.0114	upregulated	-1.03552	0.01245
ENSBTAG00000020676	MMP9	B	C	4.15276	2.30634	upregulated	-0.848466	0.0126
ENSBTAG00000020872	C5AR1	B	C	69.9562	46.6524	upregulated	-0.5845	0.01595
ENSBTAG00000027051	PTAFR	B	C	30.2115	21.8814	upregulated	-0.465393	0.04115
ENSBTAG00000031851	SELPLG	B	C	178.132	130.633	upregulated	-0.447432	0.0489

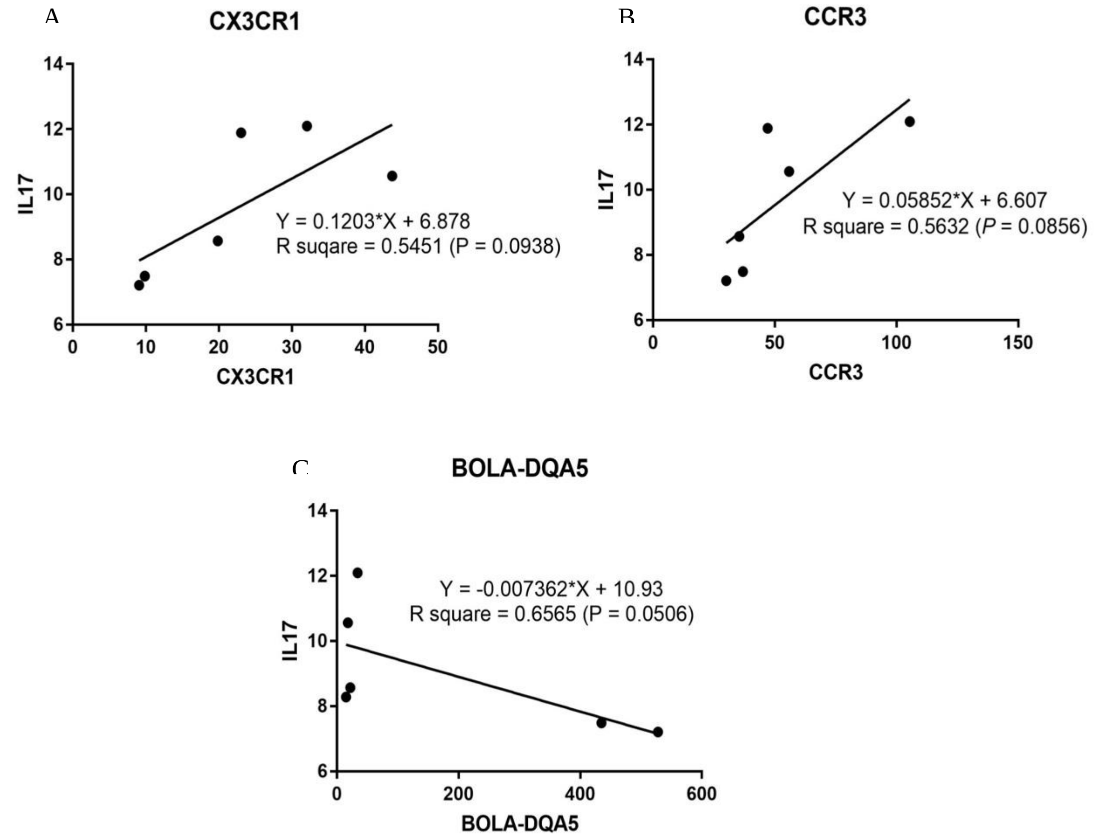

Supplementary File 8. The linear regression analyses between serum IL-17 and the expression levels of *CX3CR1* (A), *CCR3* (B), and *BOLA-DQA5* (C).
